# Outcomes and costs of remote patient monitoring among patients with implanted cardiac defibrillators: An economic model based on the PREDICT RM database

**DOI:** 10.1111/jce.13934

**Published:** 2019-04-29

**Authors:** James P. Hummel, Robert J. Leipold, Stacey L. Amorosi, Haikun Bao, Kristen A. Deger, Paul W. Jones, Anuraag R. Kansal, Lesli S. Ott, Sean Stern, Kenneth Stein, Jeptha P. Curtis, Joseph G. Akar

**Affiliations:** ^1^ Division of Cardiology University of Wisconsin School of Medicine and Public Health Madison Wisconsin; ^2^ Evidera Bethesda Maryland; ^3^ Boston Scientific Corporation Marlborough Massachusetts; ^4^ Yale University School of Medicine and Center for Outcomes Research and Evaluation, Yale‐New Haven Hospital New Haven Connecticut; and on behalf of the NCDR

**Keywords:** cost‐effectiveness, implantable cardioverter‐defibrillators, remote monitoring

## Abstract

**Background:**

Remote monitoring of implantable cardioverter‐defibrillators has been associated with reduced rates of all‐cause rehospitalizations and mortality among device recipients, but long‐term economic benefits have not been studied.

**Methods and Results:**

An economic model was developed using the PREDICT RM database comparing outcomes with and without remote monitoring. The database included patients ages 65 to 89 who received a Boston Scientific device from 2006 to 2010. Parametric survival equations were derived for rehospitalization and mortality to predict outcomes over a maximum time horizon of 25 years. The analysis assessed rehospitalization, mortality, and the cost‐effectiveness (expressed as the incremental cost per quality‐adjusted life year) of remote monitoring versus no remote monitoring. Remote monitoring was associated with reduced mortality; average life expectancy and average quality‐adjusted life years increased by 0.77 years and 0.64, respectively (6.85 life years and 5.65 quality‐adjusted life years). When expressed per patient‐year, remote monitoring patients had fewer subsequent rehospitalizations (by 0.08 per patient‐year) and lower hospitalization costs (by $554 per patient year). With longer life expectancies, remote monitoring patients experienced an average of 0.64 additional subsequent rehospitalizations with increased average lifetime hospitalization costs of $2784. Total costs of outpatient and physician claims were higher with remote monitoring ($47 515 vs $42 792), but average per patient‐year costs were lower ($6232 vs $6244). The base‐case incremental cost‐effectiveness ratio was $10 752 per quality‐adjusted life year, making remote monitoring high‐value care.

**Conclusion:**

Remote monitoring is a cost‐effective approach for the lifetime management of patients with implantable cardioverter‐defibrillators.

## 1 INTRODUCTION

The survival of patients at high risk of sudden cardiac arrest can be improved with the use of implantable cardioverter‐defibrillators (ICDs).^1^ The long‐term mortality and morbidity of patients who receive ICDs remain substantial, however. In addition to the physician visits needed to manage disease‐related morbidity, current guidelines recommend that patients with ICDs should be evaluated every 3 to 6 months to assess device function.^2^ This regimen can impose a considerable burden on both patients and physicians if patients must be evaluated in the office. As a consequence, device follow‐up is not reliable in routine clinical practice, with nearly one‐quarter of patients not seen in‐person within a year of device implantation.^3^

Remote patient monitoring (RPM) has been promoted as a strategy to reduce this burden. It can improve the efficiency of care delivery by replacing at least some in‐office visits with remote monitoring transmissions^4^, ^5^, ^6^, ^7^ without compromising safety.^7^, ^8^, ^9^ Remote monitoring may also improve patient satisfaction and quality of life as it entails less travel time, time off work, and interruption of patient activities. Data suggests that clinically actionable events are detected sooner with remote monitoring than with standard in‐office follow‐up,^10^ potentially allowing clinicians to act on these issues before they cause increased morbidity, hospitalizations, and costs. RPM also provides a convenient means for regular assessment of device‐related parameters, such as lead impedance and battery status, which may allow early detection of a device and lead malfunction.^11^, ^12^, ^13^, ^14^, ^15^ RPM can, therefore, enhance device safety and potentially improve clinical outcomes.^10^, ^16^, ^17^, ^18^, ^19^, ^20^

RPM was associated with lower hospitalization rates and reduced mortality in the large, real‐world PREDICT RM study,^21^ and its routine use has been endorsed by professional societies.^22^ However, while RPM is widely available, it is still not universally utilized by clinicians. In a recent U.S. study, fewer than half of ICD recipients enrolled in and activated RPM,^21^ and utilization is significantly lower in Europe. To determine whether RPM has economic benefits in addition to the associated clinical benefits and to determine the magnitude of the health and economic incentives for increased use of RPM, we developed an economic model to conduct an analysis of the clinical outcomes and costs of RPM versus no RPM from a Medicare perspective. Previous studies done over limited time horizons have shown RPM to be relatively cost‐effective,^23^, ^24^, ^25^, ^26^, ^27^ but this has not been evaluated over a lifelong time horizon.

## 2 METHODS

### 2.1 The PREDICT RM database

This study represents a collaborative effort between Boston Scientific Corporation, the American College of Cardiology Foundation (ACCF) and the Yale/New Haven Hospital Center for Outcomes Research and Evaluation. Use of the ALTITUDE database was approved by Boston Scientific Corporation. Use of the ACCF National Cardiovascular Data Registry (NCDR) ICD Registry was approved by the ICD Registry Research and Publications Committee. Institutional Review Board approval was obtained and data set linkage and analysis was approved by the Yale University School of Medicine Human Investigation Committee.

The PREDICT RM database was constructed by linking various data sources^22^ and applying a set of inclusion criteria that identified patients with an RPM‐capable device and Medicare fee‐for‐service claims. This allowed us to estimate the effects of RPM on the risks and costs of rehospitalizations and outpatient care.

The PREDICT RM database was constructed by linking four data sources: (1) the American College of Cardiology (ACC) National Cardiovascular Data Registry (NCDR) ICD Registry, (2) the Boston Scientific Corporation ALTITUDE database, (3) the Social Security Death Master File, and (4) Medicare administrative claims data.^22^, ^28^, ^29^, ^30^ The data set from the NCDR ICD Registry was limited to only those patients who had previously been linked to the Death Master File using direct identifiers (including Social Security Number) to determine vital status. Patients were included if they received an RPM‐capable device with first‐time device implantation between January 2006 and March 2010. The indirect identifiers age, sex, date of implant, and facility Medicare Provider Number were used to link the ICD Registry data to a comparably limited data set from the ALTITUDE database. Risk of rehospitalization was determined by linking the study cohort with corresponding Medicare fee‐for‐service administrative claims data for beneficiaries who were 65 or older.

### 2.2 Patient population

The patient population for this study was composed of Medicare patients with RPM‐capable devices (N = 15 254; control = 9906; RPM = 5348) taken from the population studied in the PREDICT RM database. Simulated individual‐patient profiles were created based on the categorical distributions of patient characteristics in the PREDICT RM database (Table 1). To reflect the heterogeneity of the real patient population and to better preserve correlations among patient characteristics, the patient population was stratified into subgroups based on the predicted times to rehospitalization and death—the key outcomes of interest. Details of the risk stratification (eTable 1) and patient characteristics for the risk‐stratified subgroups (eTable 2) can be found in the Appendix (“Patient Characteristics by Risk Strata”).

**Table 1:** PREDICT RM patient population characteristics

Patient characteristic	Category	Control		RPM		Total	
		n = 9906		n = 5348		n = 15 254	
RPM enrolled		2664	26.9%	5348	100.0%	8012	52.5%
Age	65‐74	4524	45.7%	2580	48.2%	7104	46.6%
	≥75	5382	54.3%	2768	51.8%	8150	53.4%
NYHA class	I/II	3348	33.8%	1651	30.9%	4999	32.8%
	III/IV	6552	66.1%	3690	69.0%	10 242	67.1%
Sex	Male	7104	71.7%	3846	71.9%	10 950	71.8%
	Female	2802	28.3%	1502	28.1%	4304	28.2%
Race	White, non‐Hispanic	8270	83.5%	4789	89.5%	13 059	85.6%
	Black, non‐Hispanic	786	7.9%	323	6.0%	1109	7.3%
	Hispanic	486	4.9%	111	2.1%	597	3.9%
	Other	352	3.6%	120	2.2%	472	3.1%
Admission reason	Admitted for this procedure	5839	58.9%	3518	65.8%	9357	61.3%
	Hospitalized, cardiac	1571	15.9%	656	12.3%	2227	14.6%
	Hospitalized, noncardiac	2118	21.4%	1054	19.7%	3172	20.8%
	Hospitalized, unknown	361	3.6%	117	2.2%	478	3.1%
CHF duration	No	1426	14.4%	762	14.2%	2188	14.3%
	<9 mo	2543	25.7%	1386	25.9%	3929	25.8%
	>9 mo	5932	59.9%	3190	59.6%	9122	59.8%
CHF hospitalization	Not hospitalized	5141	51.9%	3022	56.5%	8163	53.5%
	<6 mo ago	2856	28.8%	1334	24.9%	4190	27.5%
	>6 mo ago	1898	19.2%	977	18.3%	2875	18.8%
Atrial fibrillation/ atrial flutter		4025	40.6%	2066	38.6%	6091	39.9%
Nonischemic dilated cardiomyopathy	No	7070	71.4%	3668	68.6%	10 738	70.4%
	<9 mo ago	977	9.9%	555	10.4%	1532	10.0%
	>9 mo ago	1854	18.7%	1122	21.0%	2976	19.5%
Previous CABG/PCI		4350	43.9%	2292	42.9%	6642	43.5%
Pacemaker insertion		1535	15.5%	786	14.7%	2321	15.2%
Cerebrovascular disease		1683	17.0%	854	16.0%	2537	16.6%
Chronic lung disease		2589	26.1%	1325	24.8%	3914	25.7%
Diabetes		4015	40.5%	1958	36.6%	5973	39.2%
Hypertension		8054	81.3%	4312	80.6%	12 366	81.1%
Renal failure (dialysis)		425	4.3%	131	2.4%	556	3.6%
QRS duration (msec)	≤120	3483	35.2%	1705	31.9%	5188	34.0%
	>120	6423	64.8%	3643	68.1%	10 066	66.0%
Intraventricular conduction	Normal	2789	28.2%	1380	25.8%	4169	27.3%
	Abnormal (LBBB)	3625	36.6%	2179	40.7%	5804	38.0%
	Abnormal (RBBB)	880	8.9%	434	8.1%	1314	8.6%
	Paced	1047	10.6%	539	10.1%	1586	10.4%
	Other	1551	15.7%	810	15.1%	2361	15.5%
Creatinine level (mg/dL)	≤1.5	7449	75.2%	4117	77.0%	11 566	75.8%
	1.5–2.5	1887	19.0%	1013	18.9%	2 900	19.0%
	>2.5	555	5.6%	208	3.9%	763	5.0%
BUN level (mg/dL)	≤20	3889	39.3%	2226	41.6%	6 115	40.1%
	20–40	4618	46.6%	2465	46.1%	7083	46.4%
	>40	1379	13.9%	645	12.1%	2024	13.3%
Sodium level (mEq/L)	≤135	1687	17.0%	826	15.4%	2513	16.5%
	135–145	8070	81.5%	4459	83.4%	12 529	82.1%
	>145	123	1.2%	51	1.0%	174	1.1%
Systolic BP (mm Hg)	≤100	635	6.4%	326	6.1%	961	6.3%
	100–130	4393	44.3%	2217	41.5%	6610	43.3%
	>130	4826	48.7%	2786	52.1%	7612	49.9%
ICD type	Single chamber	1102	11.1%	420	7.9%	1522	10.0%
	Dual chamber	2737	27.6%	1334	24.9%	4071	26.7%
	Biventricular	6054	61.1%	3590	67.1%	9644	63.2%
Teaching status	COTH	277	2.8%	208	3.9%	485	3.2%
	Teaching	2782	28.1%	1412	26.4%	4194	27.5%
	Other	2466	24.9%	1298	24.3%	3764	24.7%
Pop. density (per sq. mile)	≤3000	8747	88.3%	4972	93.0%	13 719	89.9%
	>3000	1122	11.3%	356	6.7%	1478	9.7%

Abbreviations: BP, blood pressure; BUN, blood urea nitrogen; CABG, coronary artery bypass grafting; CHF, coronary heart failure; COTH, Council of Teaching Hospitals and Health Systems; ICD, implantable cardioverter‐defibrillator; LBBB, left bundle branch block; NYHA, New York Heart Association; PCI, percutaneous coronary intervention; RBBB, right bundle branch block; RPM, remote patient monitoring.

### 2.3 Economic model

**Figure 1:**
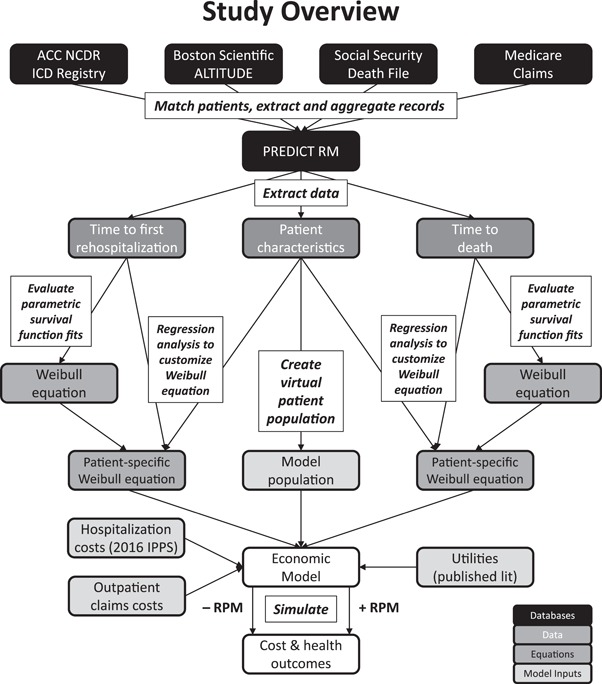
Overview of the model development process (outpatient claims not shown). The figure illustrates the data sources and inputs used in the economic model, and how the final inputs were derived. Boxes next to or over arrows describe the process completed to map PREDICT RM data to model inputs

An economic model was developed using Microsoft Excel to simulate individual patients using a time‐to‐event approach to evaluate the clinical outcomes and costs of RPM from a United States (US) Medicare perspective (Figure 1).

The model simulates hospitalization and all‐cause death events during the follow‐up period of PREDICT RM and projects those risks beyond the study period to assess the different clinical, quality of life, and cost implications of RPM versus no RPM. The model assesses the number of hospitalizations, outpatient claims, cardiovascular deaths, life years (LYs), quality‐adjusted life years (QALYs), and costs with and without RPM. Patients were followed over their lifetimes, assuming a maximum time horizon of 25 years. Costs and benefits were discounted at 3% per year.

#### 2.3.1 Model structure

The model creates simulated patients based on patient characteristic distributions from PREDICT RM. Simulated patients are then cloned and assigned to a treatment (RPM or no RPM). Patients are at risk of two major events—rehospitalization (all hospitalizations after initial device implantation) and death—while experiencing regular outpatient care.

A patient's utility is a function of baseline and accumulated comorbidities from hospitalizations in addition to the patient being hospitalized. When a patient has a rehospitalization event, comorbidity and length of stay are assigned. These factors are used to estimate a patient's quality of life.

Rehospitalization and outpatient visit rates are initially assigned based on patient characteristics and risk stratification categories, respectively. After the first rehospitalization, future rehospitalization and outpatient visit rates are assigned based solely on treatment arm.

Simulated patients continuously accumulate costs and health outcomes for rehospitalization events, outpatient claims, and accumulated comorbidities until death or the end of the time horizon. Both cost and outcomes were discounted at 3.0%.^31^

To efficiently simulate RPM in ICD patients, the model used Discretely Integrated Condition Event (DICE) simulation.^32^ The DICE modeling technique conceptualizes the decision‐analytic problem in terms of conditions (aspects of patients or the management of their health that persist over time) and events (things that happen at points in time). Conditions in the model included patient demographics, risk factors, and comorbidities; events included rehospitalizations, death, and outpatient claims. The model simulated individual patients by tracking how their conditions evolved over time and as events occur. The evolving conditions, in turn, influenced the risk of a patient experiencing future events.

#### 2.3.2 Model inputs: Clinical

##### Parametric survival equations

Kaplan‐Meier survival curves were generated for first rehospitalization and death; extrapolation beyond the observation period of the database study was achieved using parametric survival fits to the Kaplan‐Meier curves. Construction of the survival curves and the time‐to‐rehospitalization and time‐to‐death analyses are described in the Appendix (“Survival Curves for Time‐to‐rehospitalization and Time‐to‐death Analysis”).

##### Regression analysis of predictors

The survival fitting yielded distributions for each time‐to‐event curve for the overall population; the fits were then adjusted to account for individual patient characteristics. Times to first rehospitalization and death were modified based on patient characteristics and, for mortality, history of the first rehospitalization. Model building with predictors is described in detail in the Appendix (“Model Building with Predictors”).

##### Rates of subsequent rehospitalizations

The model accounts for rehospitalization events subsequent to a patient's first rehospitalization using a constant rate. Counts of second and additional rehospitalizations were divided by the duration of follow‐up (counting from the time of the first rehospitalization) to give subsequent rehospitalization rates by treatment arm (RPM vs no RPM).

##### Rates of outpatient claims

The model accounts for three classes of outpatient claims: hospital outpatient claims, ambulatory surgical center (ASC) claims, and physician claims. ASC claims constituted less than 0.6% of the total, however, and so were combined with hospital outpatient claims.

For each type of outpatient claim, rates were calculated separately before and after the first rehospitalization as the number of unique claims per patient divided by the appropriate average time—time to first rehospitalization or time from the first rehospitalization until death.

#### 2.3.3 Model inputs: Economic

##### Costs

Each hospitalization event was assigned an average cost based on the distribution of diagnosis‐related groups (DRGs) codes in the observed hospitalization events and the associated DRG costs. Outpatient claim costs were specific to the type of claim, the treatment arm, and whether the claim occurred before or after the first rehospitalization. Additional details regarding the calculation of hospitalization and outpatient claim costs can be found in the Appendix (eTable 3).

##### Utilities

Utility values for the patients are dependent on a patient's baseline characteristics, comorbidities, and rehospitalization.^30^ Patients were assumed to have a utility of 0 for their assigned length of stay during rehospitalization events. The effects of both patient characteristics (eTable 4) and comorbidities (eTable 5) were included in the estimation of patient utility.

#### 2.3.4 Model validation

To verify that the model would reproduce the observed results upon which the model inputs were based, we simulated two cohorts of patients, one with RPM and one without, and compared the model outputs to the observed data from the PREDICT RM database.

#### 2.3.5 Sensitivity and scenario analyses

With other studies failing to find an improvement in mortality due to use of RPM, complementary scenario analyses assessed the effects of patient characteristics and the importance of RPM effects on mortality as well as first rehospitalization. One‐way deterministic sensitivity analyses were conducted to investigate the sensitivity of model results to uncertainty in the values of input parameters. Additional analyses focused on the results at shorter time horizons, RPM effects on outpatient claim rates and costs, and the effects of assumptions regarding utilities.

## 3 RESULTS

### 3.1 Validation of time‐to‐event model equations

The fitting of parametric survival functions to the Kaplan‐Meier curves resulted in the selection of Weibull functions for both time to first rehospitalization and time to death. Weibull fits were selected based on a review of the Akaike and Bayesian information criteria and visual inspection of how well the fits matched the observed data (eTable 6). Extrapolations of the Weibull fits beyond the observed data were evaluated by visual inspection and judged to be clinically plausible (eFigure 1). To verify that the survival curves were properly implemented in the economic model, we compared the survival predicted by the economic model to the survival curves described by the regression equations (eFigure 2).

Table 2 shows the results of the regression analysis, including equation coefficients for all predictors that remained significant in the final analysis. RPM was found to have a coefficient of 0.0968 for time to the first rehospitalization, corresponding to a roughly 10% extension in time to rehospitalization with RPM. The RPM coefficient for time to death was 0.1666, corresponding to an approximately 18% extension in survival, all other characteristics (including rehospitalization) being equal. The large, negative coefficient (−1.545) for rehospitalization in the mortality equation is significant given that virtually all death events occurred after the first rehospitalization. RPM has, therefore, both a direct effect on mortality and an indirect effect on mortality by delaying the time to rehospitalization.

**Table 2:** Coefficients for time‐to‐event regression equations

		Time to first rehospitalization			Time to death		
Term	Value	Coefficient	SE	*P*	Coefficient	SE	*P*
Scale		1.2024	0.0128		0.9875	0.0146	
Weibull shape		0.8316	0.0088		1.0127	0.015	
Intercept		7.5446	0.0583	<0.0001	9.976	0.1219	<0.0001
RPM		0.0968	0.0307	0.0016	0.1666	0.0346	<0.0001
Age	≥75	−0.1822	0.0305	<0.0001	−0.3385	0.0339	<0.0001
NYHA class	III/IV	−0.1446	0.0355	<0.0001	−0.2279	0.0438	<0.0001
Sex	Female	−0.1257	0.0342	0.0002	0.1613	0.0382	<0.0001
Hospitalization during follow‐up					−1.5451	0.0652	<0.0001
Race	Black non‐Hispanic	−0.2122	0.0596	0.0004	−0.1957	0.0597	0.001
	Hispanic	−0.1508	0.0762	0.0478	0.1268	0.0839	0.1307
	Other	0.0143	0.085	0.8663	−0.0258	0.0896	0.7733
Admission reason	Hospitalized, cardiac	−0.2144	0.0454	<0.0001	−0.2046	0.0433	<0.0001
	Hospitalized, noncardiac	−0.2108	0.0392	<0.0001	−0.1265	0.0419	0.0026
	Hospitalized, unknown	−0.2999	0.0913	0.001	−0.255	0.0812	0.0017
CHF duration	<9 mo	0.0171	0.0535	0.7494	–	–	–
	>9 mo	−0.1339	0.0485	0.0057	–	–	–
Prior CHF hospitalization	Yes, within 0–6 mo	–	–	–	−0.2303	0.0385	<0.0001
	Yes, >6 mo ago	–	–	–	−0.1616	0.043	0.0002
Flutter		−0.183	0.0312	<0.0001	−0.1895	0.033	<0.0001
Nonischemic dilated cardiomyopathy	Yes, within past 9 mo	0.1899	0.057	0.0009	0.1951	0.0651	0.0027
	Yes, >9 mo	0.1076	0.043	0.0123	−0.0625	0.047	0.1834
CABG/PCI		−0.0885	0.034	0.0093	−0.1427	0.0369	0.0001
Permanent pacemaker		−0.1441	0.0543	0.008	0.1533	0.0587	0.009
CV disease		−0.127	0.0392	0.0012	−0.155	0.0399	0.0001
Lung disease		−0.3403	0.0335	<0.0001	−0.2539	0.0344	<0.0001
Diabetes		−0.1414	0.031	<0.0001	−0.1304	0.0334	<0.0001
Hypertension					0.0784	0.0418	0.0605
Dialysis		−0.2753	0.0921	0.0028	−0.1688	0.0783	0.0312
QRS duration	>120 msec				0.1117	0.0447	0.0124
Intraventricular conductance	Abnormal‐LBBB	0.1806	0.0399	<0.0001	−0.0131	0.0536	0.8066
	Abnormal‐RBBB	−0.0226	0.0576	0.6947	−0.1856	0.066	0.0049
	Paced	0.1374	0.0685	0.045	−0.0706	0.0789	0.3706
	Other	−0.0421	0.0473	0.3726	−0.1724	0.054	0.0014
Creatinine	1.5‐2.5 mg/dL	−0.2105	0.0431	<0.0001	−0.1641	0.043	0.0001
	>2.5 mg/dL	−0.392	0.0879	<0.0001	−0.5601	0.075	<0.0001
BUN	20‐40 mg/dL	−0.1701	0.0337	<0.0001	−0.3104	0.0402	<0.0001
	>40 mg/dL	−0.4382	0.0588	<0.0001	−0.5614	0.0582	<0.0001
Sodium	≤135 mEq/L	−0.1765	0.0406	<0.0001	−0.289	0.0393	<0.0001
	>145 mEq/L	0.0376	0.1516	0.8043	−0.2652	0.1375	0.0538
Systolic BP	100–130 mm Hg	–	–	–	0.1389	0.0607	0.0221
	>130 mm Hg	–	–	–	0.2658	0.0617	<0.0001
ICD type	Dual chamber	–	–	–	0.1859	0.0605	0.0021
	Biventricular	–	–	–	0.2123	0.0617	0.0006
Teaching status	Teaching	–	–	–	−0.0483	0.045	0.2833
	Other	–	–	–	−0.13	0.0385	0.0007
Population density	>3000/sq mi	−0.1302	0.0501	0.0094	–	–	–

Abbreviations: BP, blood pressure; BUN, blood urea nitrogen; CABG, coronary artery bypass grafting; CHF, coronary heart failure; CV, cardiovascular; ICD, implantable cardioverter‐defibrillator; LBBB, left bundle branch block; NYHA, New York Heart Association; PCI, percutaneous coronary intervention; RBBB, right bundle branch block; RPM, remote patient monitoring; SE, standard error.

### 3.2 Event rates

Event rates were calculated for rehospitalizations following the first rehospitalization and for all types of outpatient claims. The rate of subsequent rehospitalizations was different in the RPM (1.65 per year) and no‐RPM arms (1.79 per year). As with first rehospitalizations, RPM showed a benefit compared to no RPM.

The rates of hospital outpatient/ASC claims and physician claims were consistently higher in the RPM arm than they were in the no‐RPM arm. This was true before and after the first rehospitalization, and it was true for the overall population as well as for most of the risk‐stratified bins of patients (eTable 7).

**Figure 2:**
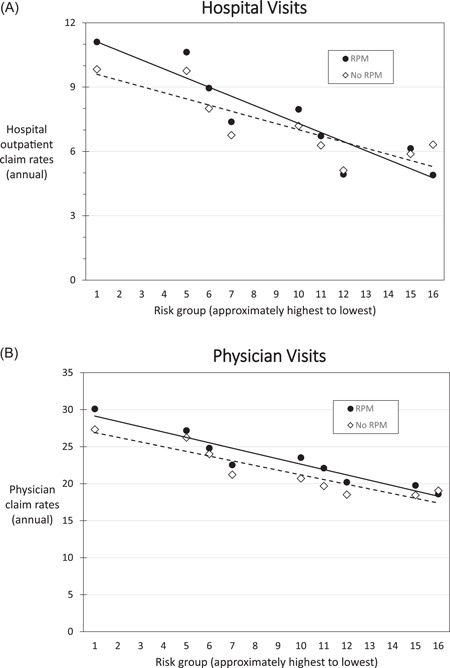
Annual outpatient claim rates by risk group. Rates for groups of fewer than 100 patients have been omitted. Lines have been added to show trends. Risk groups created by combinations of categorical rehospitalization and mortality risks are listed sequentially from highest to lowest. A linear trendline was added to illustrate observed trends in the data. For both hospital outpatient and physician claim rates, a similar trend is observed with higher rates for RPM vs no RPM, RPM, remote patient monitoring

We also investigated whether the number of outpatient claims varied by patient risk. We assigned a risk rank to each risk stratum based on the combination of baseline risks of rehospitalization and death (see Appendix (eTable 1)). Plotting the mean outpatient claim rates by risk ranking showed a clear trend toward lower outpatient claim rates in the lower‐risk groups (Figure 2).

### 3.3 Cost‐effectiveness analysis: Base case

Base‐case results are presented in Table 3. The results show clinical benefits for RPM relative to no RPM associated with a moderate increase in costs. Over the cohort's lifetime (maximum 25 years), no RPM resulted in an average of 6.85 LYs, 5.65 QALYs, and total costs of $99 815 per patient (all values discounted). No‐RPM patients had an average of 10.4 rehospitalization events and 262 outpatient unique claims throughout their follow‐up, which translated to 1.26 rehospitalization events and 32 outpatient unique claims per patient‐year (PPY). RPM resulted an increase in both LYs (7.62) and QALYs (6.29) with higher total costs ($106 729). Patients receiving RPM had both higher rehospitalization counts (11.0) and outpatient unique claims (317); however, the greater number of rehospitalizations was due to RPM patients living longer and thus having a longer time at risk to incur these events. With RPM, patients experienced only 1.18 rehospitalization events PPY, a reduction of 0.08 rehospitalization events PPY from the no‐RPM case.

**Table 3:** Base‐case results

Outcome	No RPM	RPM	Difference
LYs	6.85	7.62	0.77
QALYs	5.65	6.29	0.64
Rehospitalizations	10.4	11.0	0.6
PPY	1.26	1.18	−0.08
Hospital outpatient/ASC claims	68.1	84.6	16.4
Physician claims	194	233	39
Total outpatient claims	262	317	55
PPY	31.7	34.0	2.2
Rehospitalization costs	$57 023	$59 214	$2191
PPY	$8321	$7767	‐$554
Nonhospital cost	$42 792	$47 515	$4723
PPY	$6244	$6232	‐$12
Total costs	$99 815	$106 729	$6914
PPY	$14 566	$13 999	‐$566
Incremental cost per LY gained			$8966
Incremental cost per QALY gained			$10 752

Abbreviations: ASC, ambulatory surgical center; LY, life‐year; PPY, per patient‐year; QALY, quality‐adjusted life‐year; RPM, remote patient monitoring

When RPM is compared with no RPM, RPM was associated with an incremental gain of 0.64 QALYs and an increase in costs of $6914, resulting in an incremental cost‐effectiveness ratio (ICER) of $10,752/QALY.

### 3.4 Cost‐effectiveness analysis: Scenario and sensitivity analyses

Key scenarios in the analysis were those that removed specific RPM effects from the RPM‐arm projections (Table 4). When the RPM effect on survival was removed, the RPM arm dominated the no‐RPM arm (RPM was less costly and more effective). RPM still reduced the number of rehospitalizations, which indirectly reduced mortality. However, without the additional direct RPM effect on mortality, RPM and non‐RPM patients enjoyed similar life expectancies, and the additional costs associated with increased longevity were not accrued. When the RPM effect on the risk of the first rehospitalization was removed, the ICER increased 29%. Patients were still living longer with RPM but without the benefit of reduced rehospitalization rates. Results were also examined for each of the different risk strata and key baseline characteristics of interest. The most‐severe risk strata (high projected risks of both rehospitalization and death) had much shorter life expectancies than did patients in other risk strata, which resulted in lower incremental QALYs and a higher ICER than in the base case.

**Table 4:** Results of scenario analyses

Scenario	Incremental QALYs	Incremental costs	ICER
Base case	0.64	$6914	$10 752
Each risk‐stratified subpopulation individually			
D1‐R1	0.33	$4998	$15 275
D2‐R1	0.47	$5452	$11 492
D3‐R1	0.71	$5829	$8262
D1‐R2	0.43	$15 044	$35 198
D2‐R2	0.59	$7803	$13 241
D3‐R2	0.69	$8525	$12 275
D4‐R2	0.71	$17 109	$24 263
D2‐R3	0.65	$6730	$10 303
D3‐R3	0.71	$6783	$9493
D4‐R3	0.72	$4419	$6170
D2‐R4	0.66	$14 442	$21 772
D3‐R4	0.72	$2354	$3270
D4‐R4	0.72	−$4066	Dominant
Patient characteristics			
AF = 100%	0.60	$7211	$11 967
Cerebrovascular disease = 100%	0.57	$7210	$12 539
Chronic lung disease = 100%	0.57	$6756	$11 901
Diabetes = 100%	0.61	$7123	$11 760
Hypertension = 100%	0.64	$6926	$10 853
RPM coeff = 0, rehospitalization	0.55	$7650	$13 914
RPM coeff = 0, death	0.12	−$3734	Dominant
RPM coeff = 0, rehospitalization and death	0.02	−$3303	Dominant
Time horizon			
Time horizon = 2	0.03	−$768	Dominant
Time horizon = 5	0.13	−$635	Dominant
Time horizon = 10	0.30	$1058	$3573
Costs and resource use			
Death cost = DRG 283 (MI Death) cost	0.64	$6619	$10 294
LoS equivalent (no RPM value), first rehospitalization and subsequent rehospitalization	0.64	$6829	$10 729
Equal hospitalization outpatient/ASC costs (no RPM value), before and after rehospitalization	0.64	$10 003	$15 555
Equal physician visit costs (no RPM value), before and after rehospitalization	0.64	$6884	$10 705
Equal hospitalization outpatient/ASC rates (no RPM value), before and after rehospitalization	0.64	$4470	$6951
Equal physician visit rates (no RPM value), before and after rehospitalization	0.64	$5849	$9094
Equal subsequent rehospitalization rates (no RPM value)	0.63	$11 447	$18 102
Utility			
No utility of zero during hospitalization	0.64	$6914	$10 790
No disutility following hospitalization	0.66	$6914	$10 467
Two lines above combined	0.66	$6914	$10 504

Abbreviations: AF, atrial fibrillation; ASC, ambulatory surgical center; DRG, diagnosis‐related group; ICER, incremental cost‐effectiveness ratio; LoS, length of stay; MI, myocardial infarction; QALY, quality‐adjusted life‐year; RPM, remote patient monitoring

Changes that occurred in hospital/ASC outpatient claims before a patient's first rehospitalization had a greater effect than proportionally similar changes in hospital/ASC outpatient claims after rehospitalization or any physician claims. The main driver of results—rehospitalizations—had generally proportional effects on results when varied by 20% (eTable 8). Details of these and other scenarios and sensitivity analyses are described in the Appendix (“Scenario and Sensitivity Analysis”).

## 4 DISCUSSION

The clinical utility of remote monitoring in ICD patients is well established and endorsed in the Heart Rhythm Society/European Heart Rhythm Association (HRS/EHRA) Expert Consensus Statement.^33^ However, the cost‐effectiveness of remote monitoring is less well established and may contribute to the underutilization of RPM. This study utilizes an economic model to analyze the cost‐effectiveness of remote monitoring of ICDs over a lifelong time horizon based on Medicare claims data. The following are the main findings.

1) RPM is associated with improved survival, with an increase in discounted life expectancy of 9.3 months (0.77 LYs) and in discounted quality‐adjusted life expectancy of 7.7 months (0.64 QALYs).
2) Although total costs and the number of rehospitalizations are increased due to improved survival, the number of rehospitalizations and overall costs PPY decrease with RPM.
3) With only a direct effect of RPM on the hospitalization rate, RPM becomes a cost‐saving strategy that still provides health benefits above that of no‐RPM.
4) The incremental cost‐effectiveness ratio for remote monitoring was $10 752 per QALY gained, making RPM “high‐value” care by the ACC/American Heart Association (AHA) criterion (<$50 000/QALY).^34^
5) These results were robust to various sensitivity and scenario analyses.

Previous cost‐effectiveness analyses from small randomized studies have shown RPM to be cost saving or neutral compared with conventional in‐office follow‐up.^23^, ^27^, ^35^, ^36^ Nonhospital costs were generally lower with RPM due to fewer scheduled office visits in the RPM arm (as defined by the protocol). In these studies, the number of unscheduled visits was higher with RPM possibly related to increased detection of arrhythmias and device malfunctions. However, the total number of scheduled plus unscheduled visits was still reduced. In our study, RPM was cost‐effective despite an increase in the rate of outpatient claims. In addition to lower visit rates in these previous studies, inpatient costs were also reduced due to fewer hospitalizations and shorter lengths of stays.^35^ In addition, device cost savings were seen in the ECOST trial^37^ due to improved battery longevity. Had such cost savings been included in our analysis, RPM would have been even more cost‐effective.

Clinical equipoise remains regarding the effect of remote monitoring on mortality. Retrospective analysis of two large independent databases^19^, ^38^ both showed an association between RPM utilization and improved survival, with a graded benefit related to the level of adherence to RPM.^38^ Results from prospective randomized trials, however, have been mixed. The IN‐TIME trial^39^ demonstrated a substantial reduction in mortality in the RPM group; however, the rigorous protocol of daily RPM transmissions also resulted in increased direct patient contact. The REM‐HF trial^40^ did not demonstrate a survival benefit for RPM; however, both arms actually utilized remote monitoring to some extent, as usual care in the control arm could have included remote follow‐up every 6 months. Similarly, no benefit was seen in the MORE‐CARE trial,^9^ which replaced in‐office visits with RPM; however, this trial was not powered to detect mortality differences. As PREDICT RM is a nonrandomized database, it is possible that the observed beneficial effects on hospitalization and mortality could be secondary to confounding factors. Sensitivity analysis was, therefore, performed to evaluate the cost‐effectiveness of RPM in the absence of any survival benefit or reduction in hospitalization rate. When the beneficial effect on rehospitalization was removed, the ICER was still well within the realm of high‐value care. When the effect on survival was removed, RPM became a dominant strategy. It continued to have beneficial effects on hospitalizations and was now also cost‐saving since there was no increase in survival time during which additional costs would accrue. The cost‐effectiveness of RPM was robust across a wide range of sensitivity analyses, with ICERs well below the “high‐value care” threshold of $50,000/QALY in every risk group and in every sensitivity and scenario analysis examined.

### 4.1 Limitations of study

A key limitation of this study is that it draws primarily from an observational data source. Although this provided a large sample from which to estimate parameters, observational studies may have unobserved confounding factors that cannot be controlled for. However, estimates for all parameters in this study were controlled for a large set of patient and provider characteristics.

This study did not differentiate Medicare costs for hospitalization or outpatient claims due to cardiac conditions from those associated with other medical conditions. Therefore, the costs associated with RPM include many outpatient claims and hospitalizations unrelated to the intervention. However, as the increased costs leading to an ICER of $10 752/QALY for RPM are due to costs accrued during the extra 9.3 months of survival, the ICER would have been even more favorable if those noncardiac expenses were excluded.

In calculating the costs of hospitalizations, hospital outpatient claims, and physician claims, we used average costs. Although these averages differentiated between RPM and no RPM, it is possible that a more detailed analysis of cost differences in the two groups would lead to different and more informative cost estimates.

Patients were included in the RPM group if only a single transmission was received, but it has been shown that there is a dose‐response relationship with RPM.^38^ Including patients with only sporadic RPM may underestimate the value of regular remote monitoring.

The model used extrapolation of first rehospitalization and mortality events to assess a time horizon spanning the patient population's lifetime, which is beyond that of the PREDICT‐RM follow‐up. Although model results were robust to changes in the model time horizon, much of the health benefit is accumulated during the extrapolated period. Therefore, the conclusions regarding the benefits of RPM may not change, but the magnitude is uncertain. One potential cost saving with RPM involves improvement in battery longevity and management of the patient as the device approaches elective replacement. Without RPM, additional visits may be needed under these circumstances. As the median PREDICT RM follow‐up was only 2.5 years (interquartile range = 1.7‐3.3 years) years, very few patients reached the point of elective replacement. This effect, if modeled, would likely reduce RPM costs and improve cost‐effectiveness.

The model was based on data derived from a Medicare population and thus no patients under age 65 were included, with more than half of the cohort over 75. Thus the findings of this study may not apply to younger, healthier patients with ICDs. In addition, analyses in this manuscript were restricted to patients receiving Boston Scientific devices. Although nearly all currently used ICDs have the capacity for RPM, the technology and interfaces differ across manufacturers, and the extent to which our findings are generalizable to other devices is not known.

## 5 CONCLUSIONS

Despite its universal endorsement by professional societies, the low RPM participation rate seen in some recent clinical trials suggests that only a fraction of the potential health and economic benefits of RPM are being realized. This is likely due to a combination of factors, including the lack of long‐term cost‐effectiveness data for RPM. Using an economic model based on Medicare claims from the PREDICT RM cohort, this is the first study to assess the cost‐effectiveness of RPM over a lifetime time horizon. RPM was associated with improved survival, reduced hospitalization rates, and decreased healthcare costs PPY when compared with conventional care. Even when RPM does not have a direct effect on mortality, RPM is the preferred strategy, dominating no RPM. The ICER of $10 752/QALY gained clearly makes RPM high‐value care and underscores the importance of increasing utilization of RPM among ICD recipients.

## AUTHOR CONTRIBUTIONS

JPH, JPC, and JGA contributed to design of the study; analysis and interpretation of the data; and writing of the manuscript. HB and LSO contributed to design and conduct of study; management, analysis, and interpretation, of the data, and review of the manuscript. RJL, KAD, ARK, and SS contributed to design and conduct of study; the development of the economic model; management, analysis, and interpretation of the data; and writing and review of the manuscript. SLA, PWJ and KS contributed to the design and conduct of the study; the collection, management, analysis, and interpretation of the data; and the review and approval of the article.

## FUNDING INFORMATION

This study was sponsored by Boston Scientific Corporation.

## Supporting information

Supporting Information

## References

[1] Goldenberg I , Gillespie J , Moss AJ , et al. Long‐term benefit of primary prevention with an implantable cardioverter‐defibrillator: an extended 8‐year follow‐up study of the Multicenter Automatic Defibrillator Implantation Trial II. Circulation. 2010;122(13):1265‐1271. 20837894 10.1161/CIRCULATIONAHA.110.940148

[2] Wilkoff BL , Auricchio A , Brugada J , et al. HRS/EHRA expert consensus on the monitoring of cardiovascular implantable electronic devices (CIEDs): description of techniques, indications, personnel, frequency and ethical considerations. Heart Rhythm. 2008;5(6):907‐925. 18551743 10.1016/j.hrthm.2008.04.013

[3] Al‐Khatib SM , Mi X , Wilkoff BL , et al. Follow‐up of patients with new cardiovascular implantable electronic devices: are experts' recommendations implemented in routine clinical practice? Circulation Arrhythmia and electrophysiology. 2013;6(1):108‐116. 23264436 10.1161/CIRCEP.112.974337 PMC3640354

[4] Bikou O , Licka M , Kathoefer S , Katus HA , Bauer A. Cost savings and safety of ICD remote control by telephone: a prospective, observational study. J Telemed Telecare. 2010;16(7):403‐408. 20870684 10.1258/jtt.2010.090810

[5] Heidbuchel H , Lioen P , Foulon S , et al. Potential role of remote monitoring for scheduled and unscheduled evaluations of patients with an implantable defibrillator. Europace. 2008;10(3):351‐357. 18245771 10.1093/europace/eun010

[6] Raatikainen MJ , Uusimaa P , van Ginneken MM , Janssen JP , Linnaluoto M. Remote monitoring of implantable cardioverter defibrillator patients: a safe, time‐saving, and cost‐effective means for follow‐up. Europace. 2008;10(10):1145‐1151. 18703585 10.1093/europace/eun203 PMC2552405

[7] Varma N , Epstein AE , Irimpen A , Schweikert R , Love C , Investigators T. Efficacy and safety of automatic remote monitoring for implantable cardioverter‐defibrillator follow‐up: the Lumos‐T Safely Reduces Routine Office Device Follow‐up (TRUST) trial. Circulation. 2010;122(4):325‐332. 20625110 10.1161/CIRCULATIONAHA.110.937409

[8] Hindricks G , Elsner C , Piorkowski C , et al. Quarterly vs. yearly clinical follow‐up of remotely monitored recipients of prophylactic implantable cardioverter‐defibrillators: results of the REFORM trial. Eur Heart J. 2014;35(2):98‐105. 23868932 10.1093/eurheartj/eht207 PMC3882723

[9] Boriani G , Da Costa A , Ricci RP , et al. The MOnitoring Resynchronization dEvices and CARdiac patiEnts (MORE‐CARE) randomized controlled trial: phase 1 results on dynamics of early intervention with remote monitoring. J Med Internet Res. 2013;15(8):e167. 23965236 10.2196/jmir.2608 PMC3758044

[10] Crossley GH , Boyle A , Vitense H , Chang Y , Mead RH , Investigators C. The CONNECT (Clinical Evaluation of Remote Notification to Reduce Time to Clinical Decision) trial: the value of wireless remote monitoring with automatic clinician alerts. J Am Coll Cardiol. 2011;57(10):1181‐1189. 21255955 10.1016/j.jacc.2010.12.012

[11] Abdelhadi RH , Saba SF , Ellis CR , et al. Independent multicenter study of Riata and Riata ST implantable cardioverter‐defibrillator leads. Heart Rhythm. 2013;10(3):361‐365. 23128017 10.1016/j.hrthm.2012.10.045

[12] Hauser RG , Kallinen L. Deaths associated with implantable cardioverter defibrillator failure and deactivation reported in the United States Food and Drug Administration Manufacturer and User Facility Device Experience Database. Heart Rhythm. 2004;1(4):399‐405. 15851191 10.1016/j.hrthm.2004.05.006

[13] Hauser RG , Kallinen LM , Almquist AK , Gornick CC , Katsiyiannis WT . Early failure of a small‐diameter high‐voltage implantable cardioverter‐defibrillator lead. Heart Rhythm. 2007;4(7):892‐896. 17599673 10.1016/j.hrthm.2007.03.041

[14] Hauser RG , Maron BJ . Lessons from the failure and recall of an implantable cardioverter‐defibrillator. Circulation. 2005;112(13):2040‐2042. 16172264 10.1161/CIRCULATIONAHA.105.580381

[15] Maisel WH . Semper fidelis‐‐consumer protection for patients with implanted medical devices. N Engl J Med. 2008;358(10):985‐987. 18322280 10.1056/NEJMp0800495

[16] Klersy C , De Silvestri A , Gabutti G , et al. Economic impact of remote patient monitoring: an integrated economic model derived from a meta‐analysis of randomized controlled trials in heart failure. Eur J Heart Fail. 2011;13(4):450‐459. 21193439 10.1093/eurjhf/hfq232

[17] Klersy C , De Silvestri A , Gabutti G , Regoli F , Auricchio A. A meta‐analysis of remote monitoring of heart failure patients. J Am Coll Cardiol. 2009;54(18):1683‐1694. 19850208 10.1016/j.jacc.2009.08.017

[18] Landolina M , Perego GB , Lunati M , et al. Remote monitoring reduces healthcare use and improves quality of care in heart failure patients with implantable defibrillators: the evolution of management strategies of heart failure patients with implantable defibrillators (EVOLVO) study. Circulation. 2012;125(24):2985‐2992. 22626743 10.1161/CIRCULATIONAHA.111.088971

[19] Saxon LA , Hayes DL , Gilliam FR , et al. Long‐term outcome after ICD and CRT implantation and influence of remote device follow‐up: the ALTITUDE survival study. Circulation. 2010;122(23):2359‐2367. 21098452 10.1161/CIRCULATIONAHA.110.960633

[20] Varma N , Michalski J , Epstein AE , Schweikert R. Automatic remote monitoring of implantable cardioverter‐defibrillator lead and generator performance: the Lumos‐T Safely RedUceS RouTine Office Device Follow‐Up (TRUST) trial. Circulation Arrhythmia and electrophysiology. 2010;3(5):428‐436. 20716717 10.1161/CIRCEP.110.951962

[21] Akar JG , Bao H , Jones P , et al. Use of remote monitoring of newly implanted cardioverter‐defibrillators: insights from the patient related determinants of ICD remote monitoring (PREDICT RM) study. Circulation. 2013;128(22):2372‐2383. 24043302 10.1161/CIRCULATIONAHA.113.002481

[22] Akar JG , Bao H , Jones PW , et al. Use of remote monitoring is associated with lower risk of adverse outcomes among patients with implanted cardiac defibrillators. Circulation Arrhythmia and electrophysiology. 2015;8(5):1173‐1180. 26092577 10.1161/CIRCEP.114.003030

[23] Guedon‐Moreau L , Lacroix D , Sadoul N , et al. Costs of remote monitoring vs. ambulatory follow‐ups of implanted cardioverter defibrillators in the randomized ECOST study. Europace. 2014;16(8):1181‐1188. 24614572 10.1093/europace/euu012 PMC4114330

[24] Perl S , Stiegler P , Rotman B , et al. Socio‐economic effects and cost saving potential of remote patient monitoring (SAVE‐HM trial). Int J Cardiol. 2013;169(6):402‐407. 24383121 10.1016/j.ijcard.2013.10.019

[25] Piccini JP , Mittal S , Snell J , Prillinger JB , Dalal N , Varma N. Impact of remote monitoring on clinical events and associated health care utilization: A nationwide assessment. Heart Rhythm. 2016;13(12):2279‐2286. 27544748 10.1016/j.hrthm.2016.08.024

[26] Ricci RP , Vicentini A , D'Onofrio A , et al. Economic analysis of remote monitoring of cardiac implantable electronic devices: Results of the Health Economics Evaluation Registry for Remote Follow‐up (TARIFF) study. Heart Rhythm. 2017;14(1):50‐57. 27614025 10.1016/j.hrthm.2016.09.008

[27] Zanaboni P , Landolina M , Marzegalli M , et al. Cost‐utility analysis of the EVOLVO study on remote monitoring for heart failure patients with implantable defibrillators: randomized controlled trial. J Med Internet Res. 2013;15(5):e106. 23722666 10.2196/jmir.2587 PMC3670725

[28] Centers for Medicare & Medicaid Services (CMS) . Physician Fee Schedule FY 2016, National Payment Amount by HCPCS code. Non‐Facility Fee. 2016; https://www.cms.gov. Accessed December 12, 2016.

[29] Ghatnekar O , Bondesson A , Persson U , Eriksson T. Health economic evaluation of the Lund Integrated Medicines Management Model (LIMM) in elderly patients admitted to hospital. BMJ Open. 2013;3(1):e001563. 10.1136/bmjopen-2012-001563 PMC3553390 23315436

[30] Sullivan PW , Ghushchyan V. Preference‐Based EQ‐5D index scores for chronic conditions in the United States. Med Decis Making. 2006;26(4):410‐420. 16855129 10.1177/0272989X06290495 PMC2634296

[31] Sanders GD , Neumann PJ , Basu A , et al. Recommendations for conduct, methodological practices, and reporting of cost‐effectiveness analyses: Second panel on cost‐effectiveness in health and medicine. JAMA. 2016;316(10):1093‐1103. 27623463 10.1001/jama.2016.12195

[32] Caro JJ . Discretely integrated condition event (DICE) simulation for pharmacoeconomics. Pharmacoeconomics. 2016;34(7):665‐672. 26961779 10.1007/s40273-016-0394-z

[33] Heart Rhythm Society . 2015 HRS Expert Consensus Statement on Remote Interrogation and Monitoring for Cardiovascular Electronic Implantable Devices. 2015; http://www.hrsonline.org/Policy-Payment/Clinical-Guidelines-Documents/Expert-Consensus-on-the-Monitoring-of-Cardiovascular-Implantable-Electronic-Devices/2015-Expert-Consensus-Statement-on-Remote-Interrogation-and-Monitoring-for-CIEDs. Accessed March 30, 2017. 10.1016/j.hrthm.2015.05.008 25981148

[34] Anderson JL , Heidenreich PA , Barnett PG , et al. ACC/AHA statement on cost/value methodology in clinical practice guidelines and performance measures: a report of the American College of Cardiology/American Heart Association Task Force on Performance Measures and Task Force on Practice Guidelines. J Am Coll Cardiol. 2014;63(21):2304‐2322. 24681044 10.1016/j.jacc.2014.03.016

[35] Heidbuchel H , Hindricks G , Broadhurst P , et al. EuroEco (European Health Economic Trial on Home Monitoring in ICD Patients): a provider perspective in five European countries on costs and net financial impact of follow‐up with or without remote monitoring. Eur Heart J. 2015;36(3):158‐169. 25179766 10.1093/eurheartj/ehu339 PMC4297469

[36] Al‐Khatib SM , Piccini JP , Knight D , Stewart M , Clapp‐Channing N , Sanders GD . Remote monitoring of implantable cardioverter defibrillators versus quarterly device interrogations in clinic: results from a randomized pilot clinical trial. J Cardiovasc Electrophysiol. 2010;21(5):545‐550. 20021522 10.1111/j.1540-8167.2009.01659.x

[37] Guedon‐Moreau L , Lacroix D , Sadoul N , et al. A randomized study of remote follow‐up of implantable cardioverter defibrillators: safety and efficacy report of the ECOST trial. Eur Heart J. 2013;34(8):605‐614. 23242192 10.1093/eurheartj/ehs425 PMC3578267

[38] Varma N , Piccini JP , Snell J , Fischer A , Dalal N , Mittal S. The relationship between level of adherence to automatic wireless remote monitoring and survival in pacemaker and defibrillator patients. J Am Coll Cardiol. 2015;65(24):2601‐2610. 25983008 10.1016/j.jacc.2015.04.033

[39] Hindricks G , Taborsky M , Glikson M , et al. Implant‐based multiparameter telemonitoring of patients with heart failure (IN‐TIME): a randomised controlled trial. Lancet. 2014;384(9943):583‐590. 25131977 10.1016/S0140-6736(14)61176-4

[40] Morgan JM , Kitt S , Gill J , et al. Remote management of heart failure using implantable electronic devices. Eur Heart J. 2017;38(30):2352‐2360. 28575235 10.1093/eurheartj/ehx227 PMC5837548

