# Supplementary material for: Outcomes and costs of remote patient monitoring among patients with implanted cardiac defibrillators: An economic model based on the PREDICT RM database
Source: J Cardiovasc Electrophysiol. 2019 Apr 29;30(7):1066–77. doi: 10.1111/jce.13934 (PMC6850124; doi:10.1111/jce.13934)
Supplement: Supplementary file 1 — Supporting Information [file JCE-30-1066-s001.docx]

# Appendix. Supplemental Material

## Tables

[eTable 1. Numbers of PREDICT RM patients [and risk rank] by bin after risk stratification 2](#_Toc500263110)

[eTable 2. Characteristics by risk strata of the Medicare population from the PREDICT RM database 3](#_Toc500263111)

[eTable 3. Hospitalization costs 9](#_Toc500263112)

[eTable 4. Baseline utility equation 11](#_Toc500263113)

[eTable 5. Comorbidity disutilities 12](#_Toc500263114)

[eTable 6. Summary of survival curve fits for rehospitalization and death 14](#_Toc500263115)

[eTable 7. Annual outpatient claim rates by risk stratum before first rehospitalization and in aggregate after first rehospitalization 17](#_Toc500263116)

[eTable 8. Results of one-way (deterministic) sensitivity analyses 18](#_Toc500263117)

## Figures

[eFigure 1. Extrapolation of time-to-rehospitalization and time-to-death curves for the RPM and no-RPM populations 13](#_Toc500263118)

[eFigure 2. Comparisons of survival predicted by the economic model to fitted time-to-event distributions 16](#_Toc500263119)

## Patient Characteristics by Risk Strata

The patient population was stratified into subgroups to reflect the heterogeneity of the real patient population and to better preserve correlations among patient characteristics. As the key outcomes of interest were hospitalizations and mortality, the patient stratification was based on predicted times to first rehospitalization and predicted time to death assuming a rehospitalization, using the predictive equations for event times described below. Both predicted time to first rehospitalization and predicted time to death were partitioned into four bins, giving 16 risk groups. The rehospitalization bins were defined by predicted times to first rehospitalization of <1, 1–2, 2–3, and >3 years; the mortality bins were defined by predicted times to death (assuming a rehospitalization) of <2.1, 2.1–6.4, 6.4–12.8, and >12.8 years.

The 4 × 4 risk strata created to more accurately estimate the times-to-events resulted in 13 unique subsets of the RPM population (eTable 1; three of the “edge” risk strata did not contain any patients, and three others had 10 or fewer patients), each with their own distinct frequency of categorical characteristics. The distributions of these categorical characteristics for each risk stratum are presented in eTable 2 with the numbers of patients in each stratum at the top of each column.

To determine if outpatient claim rates depended upon a patient’s risk of rehospitalization and death, each bin was assigned a “risk rank” from 1 (highest risk) to 16 (lowest risk). The ranks were assigned by assuming that the primary determinant was the risk of death. Within each risk-of-death category, higher ranks were assigned to higher risks of rehospitalizations. Risk ranks are shown in brackets [ ] in eTable 1.

eTable 1. Numbers of PREDICT RM patients [and risk rank] by bin after risk stratification

|  | **Time to Rehospitalization (Years)** | | | | | | | |
| --- | --- | --- | --- | --- | --- | --- | --- | --- |
| **Time to death (years)** | **< 1 [R1] (highest risk)** | | **1–2 [R2]** | | **2–3 [R3]** | | **> 3 [R4]  (lowest risk)** | |
| **< 2.1 [D1]  (highest risk)** | 583 [1] | | 54 [2] | | 0 [3] | | 0 [4] | |
|  |  | (high rehospitalization risk, high death risk) | |  |  | (low rehospitalization risk, high death risk) | |  |
| **2.1-6.4 [D2]** |  |  |  |  |  |  |  |  |
|  | 1029 [5] | | 4561 [6] | | 629 [7] | | 8 [8] | |
| **6.4-12.8 [D3]** | 10 [9] | | 1269 [10] | | 2229 [11] | | 353 [12] | |
|  |  | (high rehospitalization risk, low death risk) | |  |  | (low rehospitalization risk, low death risk) | |  |
| **> 12.8 [D4]  (lowest risk)** |  |  |  |  |  |  |  |  |
|  | 0 [13] | | 9 [14] | | 180 [15] | | 235 [16] | |

eTable 2. Characteristics by risk strata of the Medicare population from the PREDICT RM database

| **Risk Stratum** | **D1-R1** | **D2-R1** | **D3-R1** | **D1-R2** | **D2-R2** | **D3-R2** | **D4-R2** | **D2-R3** | **D3-R3** | **D4-R3** | **D2-R4** | **D3-R4** | **D4-R4** |
| --- | --- | --- | --- | --- | --- | --- | --- | --- | --- | --- | --- | --- | --- |
|  | n = 583 | n = 1029 | n = 10 | n = 54 | n = 4561 | n = 1269 | n = 9 | n = 629 | n = 2229 | n = 180 | n = 8 | n = 353 | n = 235 |
| RPM enrolled | 251  (43.1%) | 505  (49.1%) | 5  (50.0%) | 22  (40.7%) | 2355  (51.6%) | 744  (58.6%) | 7  (77.8%) | 353  (56.1%) | 1384  (62.1%) | 127  (70.6%) | 4  (50.0%) | 231  (65.4%) | 177  (75.3%) |
| Age |  |  |  |  |  |  |  |  |  |  |  |  |  |
| 65-74 | 117  (20.1%) | 383  (37.2%) | 5  (50.0%) | 8  (14.8%) | 1639  (35.9%) | 788  (62.1%) | 8  (88.9%) | 241  (38.3%) | 1474  (66.1%) | 158  (87.8%) | 2  (25.0%) | 292  (82.7%) | 217  (92.3%) |
| ≥75 | 466  (79.9%) | 646  (62.8%) | 5  (50.0%) | 46  (85.2%) | 2922  (64.1%) | 481  (37.9%) | 1  (11.1%) | 388  (61.7%) | 755  (33.9%) | 22  (12.2%) | 6  (75.0%) | 61  (17.3%) | 18  (7.7%) |
| NYHA class |  |  |  |  |  |  |  |  |  |  |  |  |  |
| I/II | 57  (9.8%) | 172  (16.7%) | 6  (60.0%) | 12  (22.2%) | 1237  (27.1%) | 557  (43.9%) | 6  (66.7%) | 228  (36.2%) | 1136  (51.0%) | 120  (66.7%) | 3  (37.5%) | 190  (53.8%) | 150  (63.8%) |
| III/IV | 526  (90.2%) | 857  (83.3%) | 4  (40.0%) | 42  (77.8%) | 3324  (72.9%) | 712  (56.1%) | 3  (33.3%) | 401  (63.8%) | 1093  (49.0%) | 60  (33.3%) | 5  (62.5%) | 163  (46.2%) | 85  (36.2%) |
| Sex |  |  |  |  |  |  |  |  |  |  |  |  |  |
| Male | 498  (85.4%) | 669  (65.0%) | 1  (10.0%) | 51  (94.4%) | 3569  (78.3%) | 611  (48.1%) |  | 573  (91.1%) | 1591  (71.4%) | 56  (31.1%) | 8  (100.0%) | 294  (83.3%) | 129  (54.9%) |
| Female | 85  (14.6%) | 360  (35.0%) | 9  (90.0%) | 3  (5.6%) | 992  (21.7%) | 658  (51.9%) | 9  (100.0%) | 56  (8.9%) | 638  (28.6%) | 124  (68.9%) |  | 59  (16.7%) | 106  (45.1%) |
| Race |  |  |  |  |  |  |  |  |  |  |  |  |  |
| White, non-Hispanic | 475  (81.5%) | 795  (77.3%) | 2  (20.0%) | 46  (85.2%) | 3968  (87.0%) | 1039  (81.9%) | 6  (66.7%) | 583  (92.7%) | 2018  (90.5%) | 154  (85.6%) | 7  (87.5%) | 333  (94.3%) | 225  (95.7%) |
| Black, non-Hispanic | 66  (11.3%) | 116  (11.3%) | 2  (20.0%) | 5  (9.3%) | 315  (6.9%) | 94  (7.4%) | 1  (11.1%) | 16  (2.5%) | 106  (4.8%) | 5  (2.8%) |  | 3  (0.8%) | 2  (0.9%) |
| Hispanic | 21  (3.6%) | 90  (8.7%) | 5  (50.0%) | 3  (5.6%) | 116  (2.5%) | 103  (8.1%) | 2  (22.2%) | 2  (0.3%) | 57  (2.6%) | 14  (7.8%) |  | 3  (0.8%) | 2  (0.9%) |
| Other | 21  (3.6%) | 28  (2.7%) | 1  (10.0%) |  | 162  (3.6%) | 33  (2.6%) |  | 28  (4.5%) | 48  (2.2%) | 7  (3.9%) | 1  (12.5%) | 14  (4.0%) | 6  (2.6%) |
| Admission reason |  |  |  |  |  |  |  |  |  |  |  |  |  |
| Admitted for this procedure | 206  (35.3%) | 467  (45.4%) | 4  (40.0%) | 27  (50.0%) | 2826  (62.0%) | 830  (65.4%) | 6  (66.7%) | 527  (83.8%) | 1813  (81.3%) | 149  (82.8%) | 8  (100.0%) | 328  (92.9%) | 221  (94.0%) |
| Hospitalized, Cardiac | 203  (34.8%) | 228  (22.2%) | 1  (10.0%) | 20  (37.0%) | 673  (14.8%) | 78  (6.1%) |  | 44  (7.0%) | 78  (3.5%) | 3  (1.7%) |  | 9  (2.5%) | 3  (1.3%) |
| Hospitalized, Non-Cardiac | 122  (20.9%) | 279  (27.1%) | 4  (40.0%) | 5  (9.3%) | 945  (20.7%) | 336  (26.5%) | 2  (22.2%) | 54  (8.6%) | 328  (14.7%) | 28  (15.6%) |  | 15  (4.2%) | 11  (4.7%) |
| Hospitalized, Unknown | 52  (8.9%) | 55  (5.3%) | 1  (10.0%) | 2  (3.7%) | 117  (2.6%) | 25  (2.0%) | 1  (11.1%) | 4  (0.6%) | 10  (0.4%) |  |  | 1  (0.3%) |  |
| CHF duration |  |  |  |  |  |  |  |  |  |  |  |  |  |
| No | 20  (3.4%) | 61  (5.9%) | 1  (10.0%) | 2  (3.7%) | 549  (12.0%) | 230  (18.1%) |  | 108  (17.2%) | 527  (23.6%) | 50  (27.8%) | 2  (25.0%) | 97  (27.5%) | 64  (27.2%) |
| < 9 months | 135  (23.2%) | 149  (14.5%) | 1  (10.0%) | 23  (42.6%) | 1177  (25.8%) | 219  (17.3%) |  | 250  (39.7%) | 595  (26.7%) | 36  (20.0%) | 4  (50.0%) | 167  (47.3%) | 92  (39.1%) |
| > 9 months | 428  (73.4%) | 819  (79.6%) | 8  (80.0%) | 29  (53.7%) | 2835  (62.2%) | 820  (64.6%) | 9  (100.0%) | 271  (43.1%) | 1107  (49.7%) | 94  (52.2%) | 2  (25.0%) | 89  (25.2%) | 79  (33.6%) |
| CHF hospitalization |  |  |  |  |  |  |  |  |  |  |  |  |  |
| Not hospitalized | 139  (23.8%) | 453  (44.0%) | 9  (90.0%) | 5  (9.3%) | 2243  (49.2%) | 955  (75.3%) | 8  (88.9%) | 287  (45.6%) | 1631  (73.2%) | 162  (90.0%) | 3  (37.5%) | 249  (70.5%) | 201  (85.5%) |
| Yes,  < 6 months ago | 313  (53.7%) | 309  (30.0%) |  | 41  (75.9%) | 1370  (30.0%) | 123  (9.7%) |  | 200  (31.8%) | 253  (11.4%) | 4  (2.2%) | 5  (62.5%) | 78  (22.1%) | 25  (10.6%) |
| Yes,  > 6 months ago | 131  (22.5%) | 267  (25.9%) | 1  (10.0%) | 8  (14.8%) | 948  (20.8%) | 191  (15.1%) | 1  (11.1%) | 142  (22.6%) | 345  (15.5%) | 14  (7.8%) |  | 26  (7.4%) | 9  (3.8%) |
| Atrial fibrillation/ atrial flutter | 364  (62.4%) | 583  (56.7%) | 8  (80.0%) | 36  (66.7%) | 2075  (45.5%) | 464  (36.6%) | 3  (33.3%) | 182  (28.9%) | 450  (20.2%) | 22  (12.2%) | 2  (25.0%) | 20  (5.7%) | 11  (4.7%) |
| Non-ischemic dilated cardiomyopathy |  |  |  |  |  |  |  |  |  |  |  |  |  |
| No | 487  (83.5%) | 869  (84.5%) | 9  (90.0%) | 34  (63.0%) | 3371  (73.9%) | 946  (74.5%) | 7  (77.8%) | 334  (53.1%) | 1365  (61.2%) | 106  (58.9%) | 1  (12.5%) | 135  (38.2%) | 81  (34.5%) |
| Yes,  < 9 months ago | 14  (2.4%) | 33  (3.2%) |  | 4  (7.4%) | 302  (6.6%) | 111  (8.7%) |  | 92  (14.6%) | 336  (15.1%) | 40  (22.2%) | 4  (50.0%) | 110  (31.2%) | 99  (42.1%) |
| Yes,  > 9 months ago | 82  (14.1%) | 127  (12.3%) | 1  (10.0%) | 16  (29.6%) | 888  (19.5%) | 212  (16.7%) | 2  (22.2%) | 203  (32.3%) | 528  (23.7%) | 34  (18.9%) | 3  (37.5%) | 108  (30.6%) | 55  (23.4%) |
| Previous CABG/PCI | 381  (65.4%) | 587  (57.0%) | 5  (50.0%) | 24  (44.4%) | 2227  (48.8%) | 496  (39.1%) | 0  (0.0%) | 233  (37.0%) | 690  (31.0%) | 24  (13.3%) | 1  (12.5%) | 70  (19.8%) | 14  (6.0%) |
| Pacemaker insertion | 88  (15.1%) | 298  (29.0%) | 8  (80.0%) | 0  (0.0%) | 662  (14.5%) | 366  (28.8%) | 6  (66.7%) | 16  (2.5%) | 205  (9.2%) | 51  (28.3%) | 0  (0.0%) | 2  (0.6%) | 10  (4.3%) |
| Cerebrovascular disease | 164  (28.1%) | 300  (29.2%) | 3  (30.0%) | 10  (18.5%) | 829  (18.2%) | 184  (14.5%) | 1  (11.1%) | 55  (8.7%) | 172  (7.7%) | 8  (4.4%) | 0  (0.0%) | 14  (4.0%) | 2  (0.9%) |
| Chronic lung disease | 298  (51.1%) | 509  (49.5%) | 4  (40.0%) | 19  (35.2%) | 1215  (26.6%) | 347  (27.3%) | 0  (0.0%) | 41  (6.5%) | 174  (7.8%) | 8  (4.4%) | 0  (0.0%) | 0  (0.0%) | 2  (0.9%) |
| Diabetes | 373  (64.0%) | 596  (57.9%) | 4  (40.0%) | 18  (33.3%) | 1852  (40.6%) | 488  (38.5%) | 2  (22.2%) | 135  (21.5%) | 512  (23.0%) | 44  (24.4%) | 1  (12.5%) | 43  (12.2%) | 21  (8.9%) |
| Hypertension | 481  (82.5%) | 881  (85.6%) | 8  (80.0%) | 38  (70.4%) | 3677  (80.6%) | 1095  (86.3%) | 8  (88.9%) | 423  (67.2%) | 1769  (79.4%) | 155  (86.1%) | 4  (50.0%) | 236  (66.9%) | 185  (78.7%) |
| Renal failure (dialysis) | 122  (20.9%) | 87  (8.5%) | 1  (10.0%) | 0  (0.0%) | 59  (1.3%) | 8  (0.6%) | 0  (0.0%) | 0  (0%) | 5  (0.2%) | 0  (0.0%) | 0  (0.0%) | 0  (0.0%) | 0  (0.0%) |
| QRS duration (ms) |  |  |  |  |  |  |  |  |  |  |  |  |  |
| ≤ 120 | 175  (30.0%) | 304  (29.5%) | 2  (20.0%) | 23  (42.6%) | 1531  (33.6%) | 463  (36.5%) | 4  (44.4%) | 200  (31.8%) | 811  (36.4%) | 59  (32.8%) | 3  (37.5%) | 101  (28.6%) | 70  (29.8%) |
| > 120 | 408  (70.0%) | 725  (70.5%) | 8  (80.0%) | 31  (57.4%) | 3030  (66.4%) | 806  (63.5%) | 5  (55.6%) | 429  (68.2%) | 1418  (63.6%) | 121  (67.2%) | 5  (62.5%) | 252  (71.4%) | 165  (70.2%) |
| Intraventricular conduction |  |  |  |  |  |  |  |  |  |  |  |  |  |
| Normal | 115  (19.7%) | 259  (25.2%) | 5  (50.0%) | 9  (16.7%) | 1201  (26.3%) | 466  (36.7%) |  | 109  (17.3%) | 742  (33.3%) | 69  (38.3%) |  | 77  (21.8%) | 64  (27.2%) |
| Abnormal (LBBB) | 189  (32.4%) | 264  (25.7%) | 1  (10.0%) | 19  (35.2%) | 1651  (36.2%) | 344  (27.1%) | 5  (55.6%) | 375  (59.6%) | 973  (43.7%) | 75  (41.7%) |  | 239  (67.7%) | 154  (65.5%) |
| Abnormal (RBBB) | 85  (14.6%) | 122  (11.9%) | 1  (10.0%) | 7  (13.0%) | 445  (9.8%) | 76  (6.0%) | 2  (22.2%) | 41  (6.5%) | 124  (5.6%) | 3  (1.7%) | 6  (75.0%) | 9  (2.5%) | 3  (1.3%) |
| Paced | 47  (8.1%) | 171  (16.6%) | 3  (30.0%) | 3  (5.6%) | 504  (11.1%) | 227  (17.9%) | 1  (11.1%) | 34  (5.4%) | 163  (7.3%) | 26  (14.4%) |  | 4  (1.1%) | 5  (2.1%) |
| Other | 147  (25.2%) | 213  (20.7%) |  | 16  (29.6%) | 760  (16.7%) | 156  (12.3%) | 1  (11.1%) | 70  (11.1%) | 227  (10.2%) | 7  (3.9%) | 2  (25.0%) | 24  (6.8%) | 9  (3.8%) |
| Creatinine level (mg/dL) |  |  |  |  |  |  |  |  |  |  |  |  |  |
| ≤ 1.5 | 112  (19.2%) | 427  (41.5%) | 8  (80.0%) | 37  (68.5%) | 3549  (77.8%) | 1197  (94.3%) | 9  (100.0%) | 584  (92.8%) | 2162  (97.0%) | 175  (97.2%) | 8  (100.0%) | 350  (99.2%) | 234  (99.6%) |
| 1.5-2.5 | 274  (47.0%) | 498  (48.4%) | 2  (20.0%) | 8  (14.8%) | 941  (20.6%) | 71  (5.6%) |  | 44  (7.0%) | 65  (2.9%) | 5  (2.8%) |  | 3  (0.8%) | 1  (0.4%) |
| > 2.5 | 197  (33.8%) | 104  (10.1%) |  | 9  (16.7%) | 71  (1.6%) | 1  (0.1%) |  | 1  (0.2%) | 2  (0.1%) |  |  |  |  |
| BUN level (mg/dL) |  |  |  |  |  |  |  |  |  |  |  |  |  |
| ≤ 20 | 13  (2.2%) | 116  (11.3%) | 7  (70.0%) | 1  (1.9%) | 1313  (28.8%) | 818  (64.5%) | 8  (88.9%) | 224  (35.6%) | 1577  (70.7%) | 167  (92.8%) | 5  (62.5%) | 285  (80.7%) | 221  (94.0%) |
| 20-40 | 183  (31.4%) | 546  (53.1%) | 3  (30.0%) | 42  (77.8%) | 2874  (63.0%) | 436  (34.4%) | 1  (11.1%) | 397  (63.1%) | 649  (29.1%) | 13  (7.2%) | 3  (37.5%) | 68  (19.3%) | 14  (6.0%) |
| > 40 | 387  (66.4%) | 367  (35.7%) |  | 11  (20.4%) | 374  (8.2%) | 15  (1.2%) |  | 8  (1.3%) | 3  (0.1%) |  |  |  |  |
| Sodium level (mEq/L) |  |  |  |  |  |  |  |  |  |  |  |  |  |
| ≤ 135 | 227  (38.9%) | 218  (21.2%) | 1  (10.0%) | 23  (42.6%) | 787  (17.3%) | 115  (9.1%) |  | 72  (11.4%) | 147  (6.6%) | 3  (1.7%) | 1  (12.5%) | 21  (5.9%) |  |
| 135-145 | 349  (59.9%) | 804  (78.1%) | 9  (90.0%) | 28  (51.9%) | 3716  (81.5%) | 1152  (90.8%) |  | 540  (85.9%) | 2070  (92.9%) | 177  (98.3%) | 7  (87.5%) | 328  (92.9%) | 235  (100.0%) |
| > 145 | 7  (1.2%) | 7  (0.7%) |  | 3  (5.6%) | 58  (1.3%) | 2  (0.2%) | 9  (100.0%) | 17  (2.7%) | 12  (0.5%) |  |  | 4  (1.1%) |  |
| Systolic BP  (mm Hg) |  |  |  |  |  |  |  |  |  |  |  |  |  |
| ≤ 100 | 64  (11.0%) | 61  (5.9%) |  | 16  (29.6%) | 305  (6.7%) | 18  (1.4%) |  | 75  (11.9%) | 56  (2.5%) |  | 3  (37.5%) | 22  (6.2%) | 3  (1.3%) |
| 100-130 | 289  (49.6%) | 415  (40.3%) | 1  (10.0%) | 30  (55.6%) | 2144  (47.0%) | 416  (32.8%) | 1  (11.1%) | 324  (51.5%) | 848  (38.0%) | 37  (20.6%) | 4  (50.0%) | 178  (50.4%) | 57  (24.3%) |
| > 130 | 230  (39.5%) | 553  (53.7%) | 9  (90.0%) | 8  (14.8%) | 2112  (46.3%) | 835  (65.8%) | 8  (88.9%) | 230  (36.6%) | 1325  (59.4%) | 143  (79.4%) | 1  (12.5%) | 153  (43.3%) | 175  (74.5%) |
| ICD type |  |  |  |  |  |  |  |  |  |  |  |  |  |
| Single chamber | 66  (11.3%) | 66  (6.4%) | 1  (10.0%) | 18  (33.3%) | 486  (10.7%) | 77  (6.1%) | 1  (11.1%) | 103  (16.4%) | 229  (10.3%) | 8  (4.4%) | 1  (12.5%) | 53  (15.0%) | 12  (5.1%) |
| Dual chamber | 103  (17.7%) | 214  (20.8%) | 2  (20.0%) | 10  (18.5%) | 1077  (23.6%) | 439  (34.6%) | 2  (22.2%) | 118  (18.8%) | 773  (34.7%) | 72  (40.0%) |  | 99  (28.0%) | 89  (37.9%) |
| Biventricular | 414  (71.0%) | 749  (72.8%) | 7  (70.0%) | 26  (48.1%) | 2998  (65.7%) | 753  (59.3%) | 6  (66.7%) | 408  (64.9%) | 1227  (55.0%) | 100  (55.6%) | 7  (87.5%) | 201  (56.9%) | 134  (57.0%) |
| Teaching status |  |  |  |  |  |  |  |  |  |  |  |  |  |
| COTH | 116  (19.9%) | 339  (32.9%) | 8  (80.0%) | 6  (11.1%) | 1145  (25.1%) | 490  (38.6%) | 5  (55.6%) | 113  (18.0%) | 597  (26.8%) | 63  (35.0%) |  | 77  (21.8%) | 80  (34.0%) |
| Teaching | 145  (24.9%) | 281  (27.3%) |  | 9  (16.7%) | 1120  (24.6%) | 331  (26.1%) | 4  (44.4%) | 128  (20.3%) | 559  (25.1%) | 57  (31.7%) |  | 85  (24.1%) | 65  (27.7%) |
| Other | 322  (55.2%) | 409  (39.7%) | 2  (20.0%) | 39  (72.2%) | 2296  (50.3%) | 448  (35.3%) |  | 388  (61.7%) | 1073  (48.1%) | 60  (33.3%) | 8  (100.0%) | 191  (54.1%) | 90  (38.3%) |
| Population density (per square mile) |  |  |  |  |  |  |  |  |  |  |  |  |  |
| ≤ 3000 | 525  (90.1%) | 838  (81.4%) | 4  (40.0%) | 51  (94.4%) | 4158  (91.2%) | 1089  (85.8%) | 6  (66.7%) | 613  (97.5%) | 2103  (94.3%) | 162  (90.0%) | 8  (100.0%) | 346  (98.0%) | 227  (96.6%) |
| > 3000 | 58  (9.9%) | 191  (18.6%) | 6  (60.0%) | 3  (5.6%) | 403  (8.8%) | 180  (14.2%) | 3  (33.3%) | 16  (2.5%) | 126  (5.7%) | 18  (10.0%) |  | 7  (2.0%) | 8  (3.4%) |

Empty cells indicate that there were no patients with the associated characteristic in the risk group.

Abbreviations: BP = blood pressure; BUN = blood urea nitrogen; CABG = coronary artery bypass graft; CHF = congestive heart failure; COTH = Council of Teaching Hospitals; ICD = implanted cardiac defibrillator; LBBB = left bundle branch block; NYHA = New York Heart Association; PCI = percutaneous coronary intervention; RBBB = right bundle branch block; RPM = Remote patient monitoring.

## Costs

Hospitalization costs from the Medicare 2016 Inpatient Prospective Payment System were assigned to appropriate diagnosis-related groups (DRGs) and an overall weighted-average hospitalization cost was calculated. Separate costs were calculated for first and all subsequent hospitalizations and applied to both RPM and no-RPM hospitalizations (eTable 3). Percentages for each DRG code are shown for first and subsequent hospitalizations normalized to sum to 100%.

Four average costs were computed for each outpatient claim type: RPM vs. no RPM patients both before and after first rehospitalization. Average costs for hospital outpatient/ASC claims were calculated based on observed distributions of Healthcare Common Procedure Coding System (HCPCS) codes. The frequencies and costs of each code^1,2^ were used to calculate the four weighted-average claim costs.

Similarly, average costs for physician claims were calculated based on observed distributions of provider specialty codes. Each observed code was classified as a “general practitioner” or “specialist” code, and standard costs^3,4^ were obtained for general practitioner and cardiologist (taken to be a representative “specialist”) claims. The frequencies and costs of each class of claims were used to calculate the four weighted-average physician claim costs. An Excel® workbook showing calculation of outpatient claim costs is available upon request.

eTable 3. Hospitalization costs

| **DRG** | **ICD9** | **Comorbidity** | **DRG Payment** | **First Hospitalization** | **Subsequent Hospitalizations** |
| --- | --- | --- | --- | --- | --- |
| 637 | 250 | Diabetes mellitus | $8,164.25 | 0.4% | 0.4% |
| 638 | 250 | Diabetes mellitus | $4,998.48 | 0.9% | 0.9% |
| 640 | 259 | Other endocrine disorders | $6,684.73 | 1.1% | 1.4% |
| 641 | 259 | Other endocrine disorders | $4,264.92 | 2.3% | 1.9% |
| 811 | 289 | Other blood diseases | $7,673.44 | 0.9% | 0.5% |
| 812 | 289 | Other blood diseases | $5,062.86 | 1.0% | 1.2% |
| 250 | 410 | Acute myocardial infarction | $15,932.19 | 0.5% | 0.6% |
| 251 | 410 | Acute myocardial infarction | $9,959.76 | 1.6% | 1.1% |
| 280 | 410 | Acute myocardial infarction | $10,023.55 | 2.9% | 2.1% |
| 281 | 410 | Acute myocardial infarction | $6,043.31 | 1.1% | 0.8% |
| 282 | 410 | Acute myocardial infarction | $4,463.38 | 0.4% | 0.1% |
| 283 | 410 (dead) | Acute myocardial infarction | $9,812.10 | 0.4% | 0.4% |
| 303 | 414 | Other chronic ischemic heart diseases | $3,795.97 | 1.5% | 1.0% |
| 308 | 427 | Cardiac dysrhythmias | $7,176.13 | 2.5% | 2.1% |
| 309 | 427 | Cardiac dysrhythmias | $4,637.02 | 4.6% | 2.4% |
| 310 | 427 | Cardiac dysrhythmias | $3,312.24 | 3.9% | 2.0% |
| 127 | 428 | Heart failure | $5,733.23 | 3.1% | 1.2% |
| 189 | 428 | Heart failure | $7,244.05 | 1.0% | 1.7% |
| 291 | 428 | Heart failure | $8,746.61 | 10.9% | 14.4% |
| 292 | 428 | Heart failure | $5,733.23 | 13.6% | 17.4% |
| 293 | 428 | Heart failure | $3,979.06 | 8.5% | 7.9% |
| 219 | 429 | Ill-defined heart disease | $44,645.57 | 0.2% | 0.3% |
| 237 | 429 | Ill-defined heart disease | $3,795.97 | 0.5% | 0.5% |
| 238 | 429 | Ill-defined heart disease | $6,254.75 | 1.0% | 0.6% |
| 286 | 429 | Ill-defined heart disease | $12,860.92 | 1.2% | 0.9% |
| 287 | 429 | Ill-defined heart disease | $6,828.84 | 3.1% | 2.2% |
| 64 | 436 | CVA | $10,233.22 | 1.7% | 0.9% |
| 65 | 436 | CVA | $6,256.52 | 2.4% | 1.2% |
| 66 | 436 | CVA | $4,473.42 | 0.7% | 0.3% |
| 264 | 459 | Other circulatory disease | $16,584.83 | 0.2% | 0.6% |
| 314 | 459 | Other circulatory disease | $11,419.20 | 0.5% | 1.3% |
| 315 | 459 | Other circulatory disease | $5,742.09 | 1.1% | 0.7% |
| 316 | 459 | Other circulatory disease | $3,837.90 | 0.7% | 0.4% |
| 190 | 492 | Emphysema | $6,838.29 | 2.6% | 3.1% |
| 191 | 492 | Emphysema | $5,505.24 | 1.9% | 2.4% |
| 192 | 492 | Emphysema | $4,319.26 | 1.1% | 1.2% |
| 202 | 493 | Asthma | $5,303.84 | 0.8% | 0.5% |
| 207 | 519 | Other respiratory system diseases | $31,597.42 | 0.4% | 0.6% |
| 208 | 519 | Other respiratory system diseases | $13,616.93 | 1.4% | 1.8% |
| 391 | 530 | Diseases of esophagus | $7,043.24 | 0.7% | 0.8% |
| 392 | 530 | Diseases of esophagus | $4,370.65 | 2.7% | 2.9% |
| 371 | 537 | Other gastroduodenal diseases | $10,545.07 | 0.1% | 0.4% |
| 372 | 537 | Other gastroduodenal diseases | $6,550.06 | 0.1% | 0.6% |
| 393 | 569 | Other intestinal disorders | $9,647.91 | 0.2% | 0.6% |
| 394 | 569 | Other intestinal disorders | $5,612.15 | 0.5% | 0.7% |
| 444 | 575 | Other gallbladder disorders | $9,388.03 | 0.2% | 0.4% |
| 445 | 575 | Other gallbladder disorders | $6,232.90 | 0.2% | 0.3% |
| 682 | 586 | Renal failure nos | $8,909.62 | 2.2% | 2.8% |
| 683 | 586 | Renal failure nos | $5,555.45 | 2.9% | 3.6% |
| 684 | 586 | Renal failure nos | $3,704.42 | 0.7% | 0.4% |
| 689 | 586 | Renal failure nos | $6,391.19 | 1.0% | 1.1% |
| 690 | 593 | Other renal and ureteral Diseases | $4,623.44 | 1.3% | 2.0% |
| 698 | 593 | Other renal and ureteral diseases | $9,168.91 | 0.1% | 0.3% |
| 551 | 724 | Back disorder nec and nos | $9,197.85 | 0.5% | 0.3% |
| 552 | 724 | Back disorder nec and nos | $5,107.75 | 1.4% | 1.0% |
| 554 | 733 | Other bone and cartilage disease | $4,333.44 | 0.3% | 0.4% |
| *Weighted-Average Cost* | | | | $6,628.70 | $6,994.53 |

Abbreviations: DRG = diagnosis-related group; ICD-9 = International Classification of Diseases–Ninth Revision; CVA = acute cerebrovascular disease

## Utilities

The effects on a patient’s utility of both patient characteristics (eTable 4) and comorbidities as well as the accumulated count of unique comorbidities (eTable 5) were derived from Sullivan 2006.^5^ DRG codes from rehospitalization records from the PREDICT RM study were mapped to International Classification of Diseases—Ninth Revision (ICD-9) codes to allow assignment of associated utility decrements.

eTable 4. Baseline utility equation

| **Variable** | **Coefficient** |
| --- | --- |
| Age | -0.0007 |
| Male | 0.0007 |
| Hispanic | 0.0052 |
| Black | -0.0006 |
| Other Race | -0.0007 |
| Constant | 1.0073 |

eTable 5. Comorbidity disutilities

| **ICD-9 Code** | **Comorbidity** | **Disutility** |
| --- | --- | --- |
| ICD-9 250 | Diabetes | -0.0351 |
| ICD-9 259 | Other endocrine disorders | -0.0168 |
| ICD-9 289 | Other blood disease | -0.0359 |
| ICD-9 401 | Hypertension | -0.0250 |
| ICD-9 410 | Acute myocardial infarction (MI) | -0.0409 |
| ICD-9 414 | Ischemic heart disease/previous MI | -0.0336 |
| ICD-9 427 | Cardiac dysrhythmias | -0.0190 |
| ICD-9 428 | Heart failure | -0.0635 |
| ICD-9 429 | Ill-defined heart disease | -0.0492 |
| ICD-9 436 | Cerebrovascular disease | -0.0524 |
| ICD-9 459 | Other circulatory disease | -0.0376 |
| ICD-9 492 | Emphysema | -0.0667 |
| ICD-9 493 | Asthma | -0.0213 |
| ICD-9 518 | Chronic lung disease | -0.0428 |
| ICD-9 519 | Other respiratory system diseases | -0.0277 |
| ICD-9 530 | Diseases of esophagus | -0.0216 |
| ICD-9 537 | Other gastroduodenal diseases | -0.0311 |
| ICD-9 569 | Other intestinal disorders | -0.0193 |
| ICD-9 575 | Other gallbladder disorders | -0.0063 |
| ICD-9 586 | Renal failure/dialysis | -0.0603 |
| ICD-9 593 | Other renal and ureteral diseases | -0.0527 |
| ICD-9 724 | Back disorder nec and nos | -0.0455 |
| ICD-9 733 | Other bone and cartilage diseases | -0.0182 |
| **NCC** | **Number of Chronic Conditions (NCC)** | **Disutility** |
| NCC = 2 |  | -0.0942 |
| NCC = 3 |  | -0.0876 |
| NCC = 4 |  | -0.0711 |
| NCC = 5 |  | -0.0547 |
| NCC = 6 |  | -0.0418 |
| NCC = 7 |  | -0.0350 |
| NCC = 8 |  | -0.0344 |
| NCC = 9 |  | 0.0260 |
| NCC ≥10 |  | 0.0970 |
|  | **Outpatient Visits** | **Disutility** |
| Per visit | Duration of disutility = 1 day | -0.0028^6^ |

Abbreviations: ICD-9 = International Classification of Diseases—Ninth Revision; NCC = Number of chronic conditions

## Survival Curves for Time-to-rehospitalization and Time-to-death Analyses

The technique used to develop the parametric survival fits formally accounts for censored observations and uses statistical distributions that can account for the typically skewed distributions of time-to-event variables. The models that were considered in the fitting process were the Weibull, log-normal, log-logistic, exponential, generalized gamma, and Gompertz distributions (eTable 6). The Weibull distribution was selected as the best fit based on a combination of the Akaike Information Criterion (AIC) and Bayesian Information Criterion (BIC) and an assessment of clinical plausibility, with the assessment of clinical plausibility based primarily on the extrapolation of the survival curves to long times (eFigure 1).

eFigure 1. Extrapolation of time-to-rehospitalization and time-to-death curves for the RPM and no-RPM populations


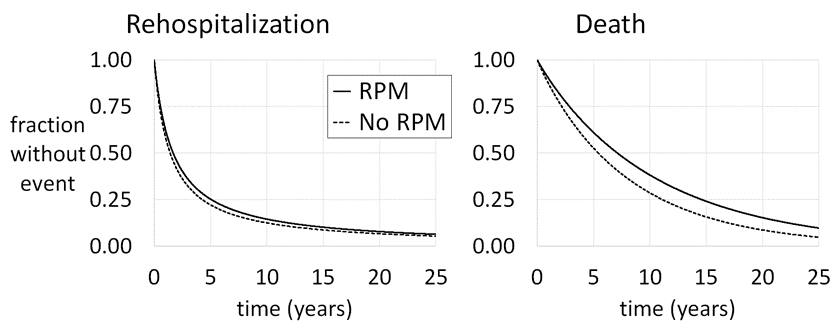


Abbreviations: RPM = remote patient monitoring

eTable 6. Summary of survival curve fits for rehospitalization and death

| ***Analysis*** | ***Intercept*** | ***SE*** | ***Scale*** | ***SE*** | ***Shape*** | ***SE*** | ***RPM**** | ***SE*** | ***AIC*** | ***BIC*** |
| --- | --- | --- | --- | --- | --- | --- | --- | --- | --- | --- |
| *Time to rehospitalization (days)* | | | | | | | | | | |
| Weibull | 6.7600 | 0.0192 | 1.2415 | 0.0133 | 0.8055 | 0.0086 | 0.1435 | 0.0313 | 33780.80 | 33802.76 |
| Log-normal | 6.2353 | 0.0239 | 1.8042 | 0.0167 |  |  | 0.1791 | 0.0378 | 33899.83 | 33921.79 |
| Log-logistic | 6.2436 | 0.0220 | 1.0092 | 0.0105 |  |  | 0.1726 | 0.0355 | 33741.25 | 33763.21 |
| Exponential | 6.7105 | 0.0153 |  |  |  |  | 0.1192 | 0.0252 | 34222.33 | 34236.97 |
| Gamma | 6.5705 | 0.0309 | 1.4744 | 0.0298 | 0.5951 | 0.0445 | 0.1613 | 0.0340 | 33713.03 | 33742.30 |
| Gompertz | 6.1608 | 0.0119 |  |  |  |  | 0.2909 | 0.0206 | ** | ** |
| *Time to death (days)* | | | | | | | | | | |
| Weibull | 7.9710 | 0.0270 | 1.0382 | 0.0158 | 0.9632 | 0.0146 | 0.2723 | 0.0361 | 25608.68 | 25631.46 |
| Log-normal | 7.9385 | 0.0325 | 1.9208 | 0.0250 |  |  | 0.3637 | 0.0431 | 25734.43 | 25757.22 |
| Log-logistic | 7.7181 | 0.0267 | 0.9619 | 0.0142 |  |  | 0.3041 | 0.0380 | 25612.78 | 25635.56 |
| Exponential | 7.9272 | 0.0192 |  |  |  |  | 0.2626 | 0.0345 | 25612.86 | 25628.05 |
| Gamma† | – | – | – | – | – | – | – | – | – | – |
| Gompertz | 7.9272 | 0.0192 |  |  |  |  | 0.2626 | 0.0345 | ** | ** |

* The RPM and no-RPM arms were modeled together in a pooled analysis with an RPM coefficient.

** The Gompertz fits were calculated with a different software package, and the AIC/BIC values were not directly comparable to those for the other fits.

† The model did not converge for the gamma time-to-death fit.

Abbreviations: AIC = Akaike Information Criterion; BIC = Bayesian Information Criterion; RPM = Remote patient monitoring; SE = standard error

For the time-to-first-rehospitalization analysis, patients who died or had a first rehospitalization within 90 days after ICD implantation were excluded from the analysis, leaving 11,149 patients. For the time-to-death analysis, patients who died within 90 days after ICD implantation were excluded from the analysis, leaving 14,703 patients. This exclusion was based on the definition of RPM use, which was enrollment and activation within 90 days of implantation. In the equation for time to death, the “rehospitalization” flag used as a predictor was set if a patient was rehospitalized during that first 90 days (assuming the patient survived to be included in the time-to-death analysis) even though that patient was excluded from the time-to-first-rehospitalization analysis. Of the 10,267 patients in the time-to-death analysis for whom the “rehospitalization” flag was set, 3,484 were rehospitalized during the first 90 days.

## Model Building with Predictors

The survival fitting yields distributions for each time-to-event curve for the overall population, which were then adjusted to account for individual patient characteristics using acceleration factors from acceleration failure time regression analyses.

In the initial analysis, univariate tests were done to identify potential predictors at a significance level of p < 0.2. Predictors that were significant in the univariate analysis were included in a multivariate model. The least-significant predictors in the multivariate model were then removed one at a time until all remaining predictors were significant at the level of p < 0.05. The final list of predictors which remained significant in the multivariate model are displayed in Table 2 along with their equation coefficients.

To verify that the survival curves were properly implemented in the economic model, we compared the survival predicted by the economic model to the survival curves described by the regression equations (eFigure 2).

eFigure 2. Comparisons of survival predicted by the economic model to fitted time-to-event distributions


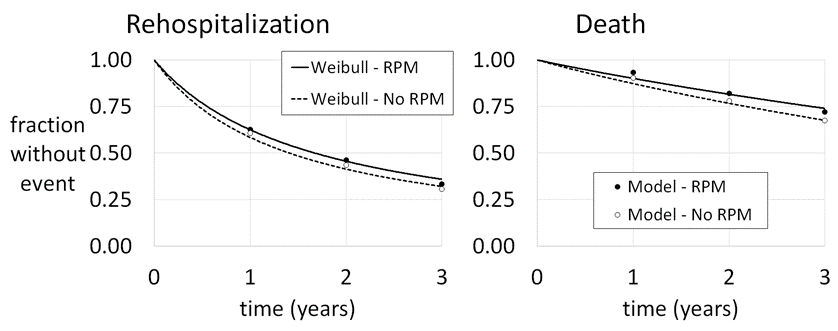


Abbreviations: RPM = remote patient monitoring

## Rates of Outpatient Claims

To avoid skewing the calculated mean rate and the standard deviation, we restricted the analysis to only patients who had Medicare coverage for at least 75% of their observation periods (6,534 patients in the no-RPM group; 3,961 patients in the RPM group). In the model, individual patients were assigned hospital outpatient/ASC and physician claim rates drawn randomly from the appropriate RPM or no-RPM distributions.

eTable 7. Annual outpatient claim rates by risk stratum before first rehospitalization and in aggregate after first rehospitalization

|  | **Hospital/ASC Outpatient Claims** | | | | **Physician Claims** | | | |
| --- | --- | --- | --- | --- | --- | --- | --- | --- |
|  | **No RPM** | | **RPM** | | **No RPM** | | **RPM** | |
|  | **Mean** | **SD** | **Mean** | **SD** | **Mean** | **SD** | **Mean** | **SD** |
| **Risk Bin** | ***Before first rehospitalization*** | | | | | | | |
| D1-R1 | 9.83 | 10.8 | 11.1 | 9.90 | 27.3 | 17.8 | 30.1 | 18.0 |
| D2-R1 | 9.75 | 12.09 | 10.63 | 10.56 | 26.2 | 20.3 | 27.2 | 16.9 |
| D3-R1 | 7.41 | 8.91 | 15.63 | 17.97 | 23.8 | 13.3 | 11.7 | 16.5 |
| D4-R1 | – | – | – | – | – | – | – | – |
| D1-R2 | 8.95 | 10.8 | 13.9 | 21.5 | 24.0 | 14.1 | 34.9 | 17.0 |
| D2-R2 | 8.00 | 9.24 | 8.95 | 9.86 | 24.0 | 17.4 | 24.8 | 15.3 |
| D3-R2 | 7.20 | 8.36 | 7.96 | 8.88 | 20.7 | 19.8 | 23.5 | 16.5 |
| D4-R2 | 4.42 | 2.48 | 8.25 | 6.68 | 14.6 | 8.2 | 23.6 | 14.4 |
| D1-R3 | – | – | – | – | – | – | – | – |
| D2-R3 | 6.76 | 8.55 | 7.38 | 8.11 | 21.2 | 14.6 | 22.5 | 13.8 |
| D3-R3 | 6.28 | 7.45 | 6.72 | 7.01 | 19.7 | 14.8 | 22.1 | 15.3 |
| D4-R3 | 5.88 | 7.27 | 6.14 | 6.79 | 18.4 | 11.4 | 19.8 | 13.3 |
| D1-R4 | – | – | – | – | – | – | – | – |
| D2-R4 | 7.63 | 3.91 | 13.8 | 9.31 | 35.7 | 21.8 | 14.8 | 5.22 |
| D3-R4 | 5.11 | 5.88 | 4.93 | 4.97 | 18.5 | 12.3 | 20.2 | 12.7 |
| D4-R4 | 6.32 | 9.53 | 4.89 | 4.60 | 19.1 | 13.6 | 18.6 | 10.2 |
|  | ***After first rehospitalization*** | | | | | | | |
| All bins | 8.81 | – | 9.67 | – | 24.3 | – | 25.7 | – |

Abbreviations: ASC = ambulatory surgical center; RPM = remote patient monitoring; SD = standard deviation

## Scenario and Sensitivity Analyses

To assess characteristics, the results for each risk-stratified subpopulation and for patients with specific clinically relevant baseline characteristics (e.g., 100% heart failure population) were assessed. In order to assess the impact of RPM clinical effects, the RPM effects on first rehospitalization and death were individually removed to test the effect on model results if only one of these effects was realized.

Across all the other risk strata, the results varied in their direction of change from the base case depending upon the balance of factors that changes across the risk strata. Results for cohorts in which all patients had, for example, heart failure, were consistent with results for high-risk patients. Changes in outpatient rates (hospital/ASC outpatient or physician claims) have the expected effect on results depending on the direction the claim rate or cost moves. Length of stay had very little effect on overall results as it affected only rehospitalization utility decrements.

eTable 8. Results of one-way (deterministic) sensitivity analyses

| **Sensitivity Analyses** | **Base-case value** | **SA value** | **Incremental QALYs** | **Incremental Costs** | **ICER** |
| --- | --- | --- | --- | --- | --- |
| Base case | — | — | 0.64 | $6,914 | $10,752 |
| Hospital Outpatient/ASC Rate | | | | | |
| No RPM - before rehospitalization | varies based on population | +20% | 0.64 | $3,440 | $5,349 |
|  |  | -20% | 0.64 | $10,388 | $16,155 |
| RPM - before rehospitalization | varies based on population | +20% | 0.64 | $10,421 | $16,207 |
|  |  | -20% | 0.64 | $3,406 | $5,297 |
| No RPM - after rehospitalization | 0.02413 | +20% (0.02896) | 0.64 | $5,040 | $7,836 |
|  |  | -20% (0.01930) | 0.64 | $8,788 | $13,667 |
| RPM - after rehospitalization | 0.02646 | +20% (0.03175) | 0.64 | $9,141 | $14,216 |
|  |  | -20% (0.02117) | 0.64 | $4,687 | $7,288 |
| Physician Visits | | | | | |
| No RPM - before rehospitalization | varies based on population | +20% | 0.64 | $5,737 | $8,921 |
|  |  | -20% | 0.64 | $8,090 | $12,583 |
| RPM - before rehospitalization | varies based on population | +20% | 0.64 | $8,240 | $12,817 |
|  |  | -20% | 0.64 | $5,587 | $8,687 |
| No RPM - after rehospitalization | 0.06644 | +20% (0.07972) | 0.64 | $4,880 | $7,587 |
|  |  | -20% (0.05315) | 0.64 | $8,948 | $13,918 |
| RPM - after rehospitalization | 0.07029 | +20% (0.08435) | 0.64 | $9,355 | $14,553 |
|  |  | -20% (0.05623) | 0.64 | $4,472 | $6,952 |
| Length of Stay | | | | | |
| No RPM - before rehospitalization | 5.87 | +20% (7.04) | 0.65 | $6,944 | $10,759 |
|  |  | -20% (4.70) | 0.64 | $6,878 | $10,735 |
| RPM - before rehospitalization | 5.40 | +20% (6.48) | 0.64 | $6,900 | $10,766 |
|  |  | -20% (4.32) | 0.65 | $6,936 | $10,749 |
| No RPM - after rehospitalization | 6.58 | +20% (7.90) | 0.66 | $7,218 | $10,869 |
|  |  | -20% (5.26) | 0.62 | $6,484 | $10,428 |
| RPM - after rehospitalization | 6.25 | +20% (7.50) | 0.62 | $6,639 | $10,673 |
|  |  | -20% (5.00) | 0.66 | $7,250 | $10,916 |
| Hospital Outpatient/ASC costs | | | | | |
| No RPM - before rehospitalization | $969.76 | +20% ($1163.72) | 0.64 | $3,440 | $5,349 |
|  |  | -20% ($775.81) | 0.64 | $10,388 | $16,154 |
| RPM - before rehospitalization | $845.14 | +20% ($1014.17) | 0.64 | $10,421 | $16,206 |
|  |  | -20% ($676.11) | 0.64 | $3,406 | $5,297 |
| No RPM - after rehospitalization | $272.87 | +20% ($327.45) | 0.64 | $5,040 | $7,837 |
|  |  | -20% ($218.30) | 0.64 | $8,788 | $13,666 |
| RPM - after rehospitalization | $261.08 | +20% ($313.30) | 0.64 | $9,141 | $14,215 |
|  |  | -20% ($208.86) | 0.64 | $4,687 | $7,288 |
| Physician visit costs | | | | | |
| No RPM - before rehospitalization | $107.62 | +20% ($129.14) | 0.64 | $5,737 | $8,922 |
|  |  | -20% ($86.09) | 0.64 | $8,090 | $12,581 |
| RPM - before rehospitalization | $107.70 | +20% ($129.24) | 0.64 | $8,240 | $12,815 |
|  |  | -20% ($86.16) | 0.64 | $5,587 | $8,688 |
| No RPM - after rehospitalization | $107.55 | +20% ($129.06) | 0.64 | $4,880 | $7,589 |
|  |  | -20% ($86.04) | 0.64 | $8,948 | $13,914 |
| RPM - after rehospitalization | $107.77 | +20% ($129.32) | 0.64 | $9,355 | $14,549 |
|  |  | -20% ($86.21) | 0.64 | $4,472 | $6,954 |
| Hospitalization Costs | | | | | |
| First hospitalization | $6,628.70 | +20% ($7,954.44) | 0.64 | $6,902 | $10,733 |
|  |  | -20% ($5,302.96) | 0.64 | $6,926 | $10,770 |
| Subsequent hospitalization | $6,994.53 | +20% ($8393.44) | 0.64 | $7,364 | $11,452 |
|  |  | -20% ($5,595.63) | 0.64 | $6,463 | $10,051 |
| Utility | | | | | |
| Baseline utility |  | +20% | 0.80 | $6,914 | $8,664 |
|  |  | -20% | 0.49 | $6,914 | $14,165 |
| Disutilities for baseline comorbidities |  | +20% | 0.63 | $6,914 | $10,973 |
|  |  | -20% | 0.66 | $6,914 | $10,539 |
| Disutilities for added comorbidities |  | +20% | 0.64 | $6,914 | $10,799 |
|  |  | -20% | 0.65 | $6,914 | $10,705 |
| NCC disutilities |  | +20% | 0.63 | $6,914 | $10,931 |
|  |  | -20% | 0.65 | $6,914 | $10,578 |
| Outpatient visit disutility |  | +20% | 0.64 | $6,914 | $10,753 |
|  |  | -20% | 0.64 | $6,914 | $10,751 |
| Rehospitalization | | | | | |
| RPM Coefficient | 0.0968 | +20% (0.11616) | 0.66 | $6,934 | $10,467 |
|  |  | -20% (0.07744) | 0.62 | $6,893 | $11,048 |
| Scale | 1.2024 | +20% (1.4429) | 0.63 | $6,999 | $11,167 |
|  |  | -20% (0.9619) | 0.65 | $7,700 | $11,809 |
| No RPM - Subsequent rehospitalization rate | 0.00490 | +20% (0.0059) | 0.67 | $(3,275) | Dominant |
|  |  | -20% (0.0039) | 0.62 | $17,199 | $27,896 |
| No RPM - Subsequent rehospitalization rate | 0.00452 | +20% (0.0054) | 0.62 | $17,859 | $28,918 |
|  |  | -20% (0.0039) | 0.67 | $(3,973) | Dominant |
| Death | | | | | |
| RPM Coefficient | 0.1666 | +20% (0.1999) | 0.75 | $9,113 | $12,153 |
|  |  | -20% (0.1333) | 0.54 | $4,891 | $9,128 |
| Experienced hospitaliza-tion during follow-up | -1.5451 | +20% (-1.8541) | 0.57 | $6,156 | $10,839 |
|  |  | -20% (-1.2361) | 0.67 | $6,688 | $9,910 |
| Scale | 0.9875 | +20% (1.1850) | 0.36 | $2,891 | $8,025 |
|  |  | -20% (0.7900) | 0.56 | $(430) | Dominant |

Abbreviations: ASC = ambulatory surgical center; DRG = diagnosis-related group; ICER = incremental cost-effectiveness ratio; LoS = length of stay; MI = myocardial infarction; NCC = number of chronic conditions; QALY = quality-adjusted life year; RPM = remote patient monitoring; SA = sensitivity analysis

## References

1. Centers for Medicare & Medicaid Services (CMS). Addendum B Updates to HCPCS Codes and OPPS. 2017; <https://www.cms.gov/Medicare/Medicare-Fee-for-Service-Payment/HospitalOutpatientPPS/Addendum-A-and-Addendum-B-Updates.html>.

2. Raatikainen MJ, Uusimaa P, van Ginneken MM, Janssen JP, Linnaluoto M. Remote monitoring of implantable cardioverter defibrillator patients: a safe, time-saving, and cost-effective means for follow-up. *Europace.* 2008;10(10):1145-1151.

3. Centers for Medicare & Medicaid Services (CMS). Physician Fee Schedule FY 2016, National Payment Amount by HCPCS code. Non-Facility Fee. 2016; <https://www.cms.gov>. Accessed December 12, 2016.

4. Centers for Medicare & Medicaid Services (CMS). Physician Fee Schedule FY 2016, National Payment Amount by HCPCS code. Non-Facility Fee. 2016; <https://www.cms.gov/>. Accessed Dec 12, 2016.

5. Sullivan PW, Ghushchyan V. Preference-Based EQ-5D index scores for chronic conditions in the United States. *Med Decis Making.* 2006;26(4):410-420.

6. Ghatnekar O, Bondesson A, Persson U, Eriksson T. Health economic evaluation of the Lund Integrated Medicines Management Model (LIMM) in elderly patients admitted to hospital. *BMJ Open.* 2013;3(1).
